# Detection of Alcoholic EEG signal using LASSO regression with metaheuristics algorithms based LSTM and enhanced artificial neural network classification algorithms

**DOI:** 10.1038/s41598-024-72926-7

**Published:** 2024-09-13

**Authors:** Gowri Shankar Manivannan, Kalaiyarasi Mani, Harikumar Rajaguru, Satish V. Talawar

**Affiliations:** 1 Malnad College of Engineering, Hassan, Karnataka India; 2 Bannari Amman Institute of Technology, Sathyamangalam, Tamilnadu India

**Keywords:** Alcoholism, Classification, EANN, EEG, LASSO regression, Computational models, Data processing, Machine learning, Diseases, Health care, Neurology, Risk factors, Biomedical engineering

## Abstract

The world has a higher count of death rates as a result of Alcohol consumption. Identification is possible because Alcoholic EEG waves have a certain behavior that is totally different compared to the non-alcoholic individual. The available approaches take longer to provide the feedback because they analyze the data manually. For this reason, in the present paper we propose a novel approach applied to detect alcoholic EEG signals automatically by using deep learning methods. Our strategy has advantages as far as fast detection is concerned; hence people can help immediately when there is a need. The potential for a significant decrease in deaths from alcohol poisoning and improvement to public health is presented by this advancement. In order to create clusters and classify the alcoholic EEG signals, this research uses a cascaded process. To begin with, an initial clustering and feature extraction is done by LASSO regression. After that, a variety of meta-heuristics algorithms like Particle Swarm Optimization (PSO), Binary Coding Harmony Search (BCHS) as well as Binary Dragonfly Algorithm (BDA) are employed for feature minimization. When this method is used, normal and alcoholic EEG signals may be differentiated using non-linear features. PSO, BCHS, and BDA features allow for estimation of statistical parameters through t-test, Friedman statistic test, Mann-Whitney U test, and Z-Score with corresponding p-values for alcoholic EEG signals. Lastly, classification is done by the use of support vector machines (SVM) (including linear, polynomial, and Gaussian kernels), random forests, artificial neural networks (ANN), enhanced artificial neural networks (EANN), and LSTM models. Results showed that LASSO regression with BDA-based EANN proposed classifier have a classification accuracy of 99.59%, indicating that our method is highly accurate at classifying alcoholic EEG signals.

## Introduction

Gulping EEG is a term that refers to the alteration of an electroencephalograph in those with Alcohol Use Disorder (AUD)^1^. Investigations have shown that continuous alcohol drinking may influence brain performance emanating in distinct EEG structures. Studies have delineated some of these peculiarities as increased alpha and beta wave activity, decrease in frontal and parietal voltage, abnormal coherence of EEG and phase-locking values^2^. These modifications are suggested to signify cognitive deficits, neural adaptation otherwise brain damage linked to heavy alcohol consumption^3^. EEG can be used as a valuable instrument for monitoring shifts occurring in the brain within relation to alcohol use disorder diagnosis and treatment. Alcohol consumption drastically affects the electrical activity of the brain which is measureable through electroencephalography (EEG)^4^. Alcohol alters the different frequency bands seen on an EEG record such as; alpha waves, beta waves theta waves and delta waves, which correspond to different cognitive processes and conscious states.

Alpha waves (8–12 Hz) are generally noticeable in passive, shut-eye conditions, and associated with decreased cortical activity. Alcohol enhances alpha wave activity as a sign of relaxation and reduced cortical activity^5^. This is consistent with the subjective feeling of being calm otherwise relaxed experienced after drinking. Beta waves (13–30 Hz) are related to active thought, problem solving, and motor activities. Alcohol boosts beta wave activity especially within the frontal cortex which may result in a subjective feeling of increased energy and sociability^6^. Theta waves (4–8 Hz) are linked to drowsiness, sleep, and cognitive functions. Alcohol reduces theta wave activity that may impair thinking and memory consolidation process. Delta waves typically have a frequency of 0. 5 − 4 Hz, are found in deep sleep stages or during unconsciousness^7^. Alcohol is known to depress delta wave activity that might result into distorted sleep patterns combined with insomnia. Other than these frequency dependent factors alcohol also influences the coherency of the EEG and phase-locking factors that suggest the interaction between different lobes of the brain. Decreased coherence, as well as phase-locking values by alcohol also implying disrupted communication among brain regions. Effects of alcohol on EEG are dose-dependent, with stimulant effects associated with low doses (rising beta and alpha activity) and depressant effects associated with high doses (falling theta and delta activity). Additionally, there are individual differences in brain chemistry, genetics, and drinking history that influence the effects of alcohol on EEG^8^. The overall role played by alcohol in the EEG is indicative of its complex nature within the brain that leads to interference on cognitive aspect, mood as well as behavior.

Recently, many researchers have examined various techniques for feature selection and extraction from EEG data in order to develop efficient algorithms for identifying alcoholism in EEG signals. In an experiment conducted by Nandini et al.^9^ the severity of alcoholism was assessed using a deep learning approach consisting of resting state EEG features and a combined CNN + LSTM model which yielded an average classification accuracy of 91% among alcoholic patients. Houchi and Lei^10^ employed hybrid models combining CNNs, LSTMs and discrete wavelet transform (DWT) to identify alcoholic EEG signals with notable achievements such as 92.77% classification accuracies when using the CNN model and 89% using LSTM model. Leila et al.^11^ came up with a unique method where principal component analysis was used for feature extraction followed by deep learning classifier based on LSTM that distinguishes between Alcoholics and Normal patients. The average accuracy obtained in this case was 93%. Shrey Agarwal et al.^12^ designed a new approach comprising Sliding Singular Spectrum Analysis based Independent Components analysis for pre-processing as well as an Artificial Neural Network (ANN) classifier resulting into a high accuracy of 97.37% while convicting Alcoholic and Non-Alcoholic EEG Signals. Rakhmatulin^13^ has proposed the classification model of Alcoholic and Non-Alcoholic EEG Signals across deep learning and machine learning approaches. Therefore, a new CNN model was introduced, and its performance was evaluated based on the results of the given EEG correlation dataset; in particular, the average classification accuracy of the proposed model reached the level of 92%.

Emad et al.^14^ proposed automatic alcoholism detection system which they used the database of the control of 45 subjects and 77 alcohol dependent subjects with each subject having 120 trials. They used Multi-Power (MP) CNN for the feature extraction of the images with classification using the Softmax classifier, a success rate of over 97% for the average classification. Zhu et al.^15^ proposed the new framework based on HVG entropy for classifying between alcoholic and normal EEG signals with a SVM classifier. Their proposed model, as tested on the dataset provided by them, recorded an average classification accuracy of 94%. The DCNN with ReLU was proposed for alcoholism detection in Hamid et al.^16^ with enough regularization and it had a promising result with an average classification accuracy of 98%. Acharya et al.^17^ proposed a new algorithm for the detection of alcoholism from EEG signals whereby the features like the approximate and sample entropy, Lyapunov exponent and higher order spectra were used. These features were used in combination with Support Vector Machine (SVM) classifiers incorporating polynomial and Radial Basis Function (RBF) kernel, the overall average classification rates were 91.7%. To compare the EEG signal, Anuragi and Sisodia^18^ proposed a technique of recognizing alcoholism by the help of Statistical features derived from the Flexibly Analytical Wavelet Transform. These features were used together with the LS-SVM polynomial kernel classifier whereby the average classification accuracy of the classifier was 99%.

Siddiqui et al.^19^ introduces a novel cost-sensitive seizure classifier based on machine leaning suitable for imbalanced EEG datasets with an accuracy of 98.47%. Using a cost-sensitive learning approach alongside a decision tree classifier has been employed in the methodology, where higher penalties are used to misclassify seizures in imbalanced datasets. Siddiqui et al.^20^ proposed a novel, fast seizure detection and localization framework that used data mining for analysis in the brain data mining on ECoG data set and had an accuracy rate of 100%. Detection speed and accuracy of the seizures are augmented by the quick identification and localization of seizures as was attributed to its use of feature extraction methods and random forest classifier. According to Siddiqui et al.^21^, different machine learning classifiers are considered in order to detect epileptic seizures. They achieve an accuracy level range of between 90% and 99% using separate methodologies. This paper looks at Support Vector Machines (SVM), Decision Trees and Neural Networks which are some of the things that need to be put into consideration while selecting characterization parameters and data preprocessing for better seizure detection performance. An optimized convolutional neural network (CNN) along with a selected feature set for epileptic seizure detection in EEG signals has been proposed by Fatma Singh and Siddiqui^22^ to achieve a percentage accuracy of 97.4%. This methodology employs two stages of feature selection before CNN classification is carried out; thus improving detection rates without compromising system speed. According to Siddiqui et al.^23^, this study was aimed at determining which type of classification technique works best when it comes to identifying epileptic seizures and its accuracy rate was reported as being within 98.3%. The authors also compare these strategies with others like SVM and KNN which are discussed together with neural networks but concentrating more on how well they handle EEG information that is meant to detect seizures.

As can be observed from the above reviewed literature, the majority of the available work employs entropy-based method, Principal Component Analysis (PCA) and other energy orientated approaches suggesting that the subject area tends to trend in this direction. This research empirically explored the effectiveness of combining LASSO regression with metaheuristic algorithms to develop an automatic and accurate alcoholism detection system using EEG signals. Given the prevalence of Artificial Neural Networks (ANN) and Long Short-Term Memory (LSTM) architectures in EEG-based studies (used in over 40% of cases), this study conducted a systematic comparison and analysis of their classification performances to determine their relative efficacy.

Deep learning’s ability to automatically extract complex features from datasets makes it an ideal tool for uncovering hidden patterns in EEG signals. While some research has applied deep learning techniques like ANN, CNN, and LSTM to diagnose alcoholism from EEG signals, there is still a need for more effective systems. This study aims to bridge that gap by proposing a novel framework that combines LASSO regression with metaheuristic algorithms and LSTM and enhanced artificial neural network (EANN) deep learning models. Our approach leverages LASSO regression for initial feature extraction, followed by metaheuristic algorithms for further feature refinement, and finally, LSTM and EANN for accurate classification. Furthermore, we employed a subject-wise k-fold cross-validation approach, where we performed 10-fold cross-validation on each individual’s EEG data and then combined the respective EEG segments. This approach helped mitigate individual variability. Unlike traditional machine learning methods, which require testing multiple classifiers to determine the most relevant features and optimal classifier, our approach streamlined this process by automatically extracting valuable features and achieving high accuracy. In this regard, our architecture is capable of learning latent features from EEG signals itself since it is free from many rigidities of traditional machine learning approaches and, therefore, yields an overwhelming classification accuracy of 99.59% and this illustrates how the model may be utilized in alcoholism diagnostic systems. This paper gives a unique method for analyzing alcoholic EEG signals, which includes:

1. LASSO regression is implemented to discover the clusters in the alcoholic EEG signals for extensive feature extraction.
2. PSO, BCHS, and BDA optimization algorithms are used in order to minimize the most significant features to obtain even better results.
3. The reduced features are then fed into appropriate LSTM and EANN models to estimate the degrees of alcoholic risk in EEG signals; this analytically serves a stable platform for analyzing and identifying alcoholism.
4. Finally, this study intends to compare and contrast the classical machine learning methods and new deep neural network-based solutions to determine the applicability of the suggested strategies.

This paper is structured as follows: “Materials and methods” describes the materials and methods used in the study focusing using LASSO on feature extraction and feature selection using various metaheuristic algorithms, followed by a description of the classification methodology in “Feature selection via different metaheuristic algorithms”. The evaluation scheme are presented in “Evaluation Scheme”, and the paper concludes in “Conclusion”, with relevant references provided at the end.

## Materials and methods

In this section includes the details about the dataset, clustering via feature extraction using LASSO Regression methodology and feature selection using various metaheuristic algorithms.

### Dataset details

**Fig. 1:**
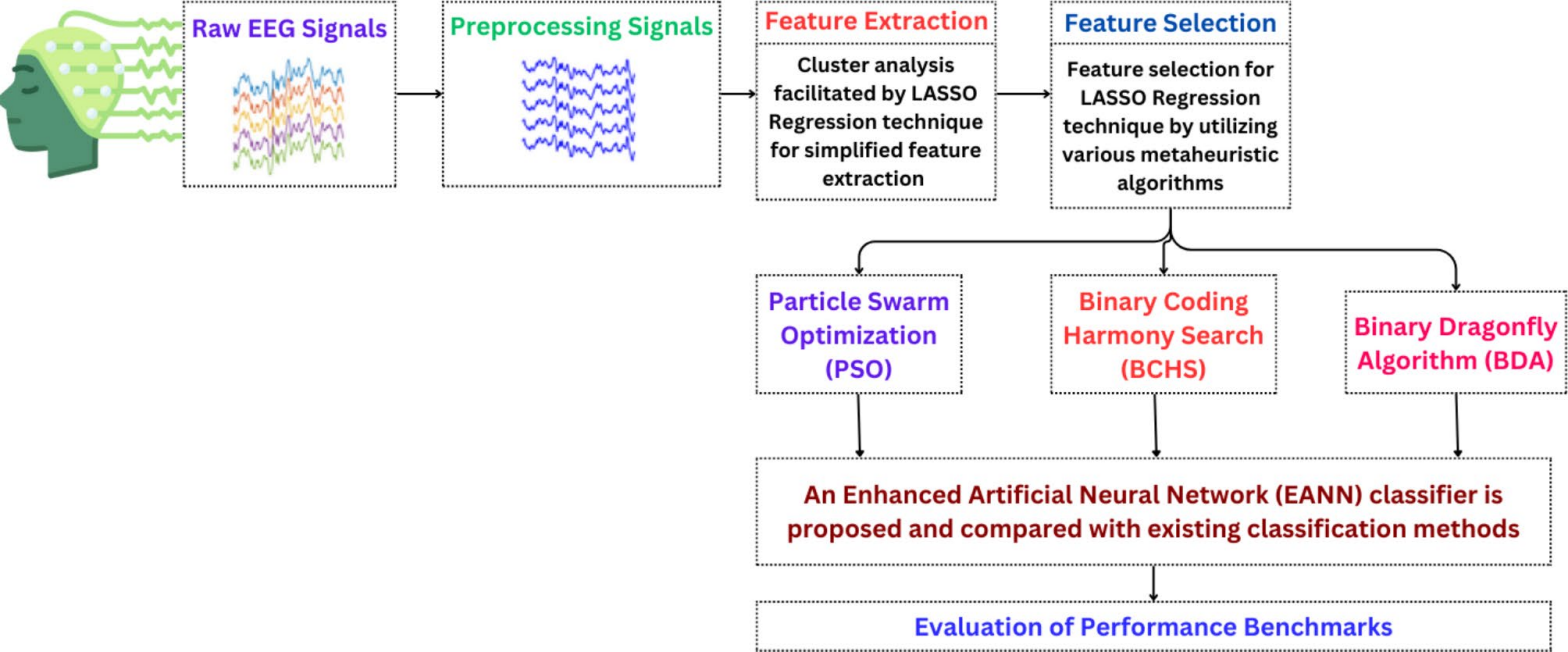
Simplified block diagram of the process.

Our study employed an alcoholic EEG dataset from the UCI KDD archive^24^, a well-known publicly available online repository at the University of California, Irvine. This dataset was chosen to explore the relationship between brain activity (reflected in EEG signals) and alcoholism. The dataset comprises EEG recordings from a total of 122 subjects (both normal and alcoholic) following the standard 10/20 International electrode placement system. Electrode impedance was maintained below 5 kΩ to ensure good signal quality. Each subject underwent 120 trials involving different stimuli, and the EEG signals were captured by 64 electrodes at a sampling rate of 256 Hz with 12-bit resolution. However, raw EEG data can be contaminated by noise from muscle movements, eye blinks, and body sway. To address this issue, we implemented a simple yet effective pre-processing technique called Independent Component Analysis (ICA) to remove these artifacts. This step is crucial because artifacts can significantly hinder the accuracy of classification algorithms. Clean EEG signals are essential for reliable alcohol level detection. Following pre-processing, relevant 1D EEG recordings for both normal and alcoholic subjects were segmented into 2D matrices using Short Time Fourier Transform (STFT)^25^ and stored in separate files, with each file containing 2560 data points. A simplified block diagram outlining our approach is provided in Fig. 1. The process involves pre-processing the EEG signals, feature extraction using LASSO Regression, feature selection using various metaheuristic algorithms like Particle Swarm Optimization (PSO), Binary Coding Harmony Search (BCHS) and Binary Dragonfly Algorithm (BDA) and finally, classification using suitable algorithms to analyze alcohol levels based on the EEG data.

### Clustering via LASSO Regression technique for simplified feature extraction

LASSO regression is also acknowledged as L1 regularization. It is a popular technique in machine learning used to estimate relationships between variables and make predictions^26^. It excels at balancing model simplicity and accuracy. LASSO achieves this by adding a penalty term to the standard linear regression model, forcing some coefficients to become exactly zero. This feature selection capability makes LASSO particularly useful for identifying and discarding irrelevant variables. The general mathematical equation for LASSO regression represented as follows^27^:

1 $$\:RSS=\lambda\:* \textit{total of each coefficient{'}s magnitude{'}s absolute value}$$

Where $\:RSS$ represents Residual Sum of Squares, it is reflecting how well the model fits the data. It measures the total squared difference between the actual values and the values predicted by the model and $\:\lambda\:$ indicates the parameter of regularization. LASSO regression is explained in detail step-by-step below:

#### Step 1

LASSO starts with a standard linear regression model assuming a linear relationship between features and the target variable. The standard linear regression equation for this case is as follows.

2 $$\:g=\:{\beta\:}_{0}+{\beta\:}_{1}{s}_{1}+{\beta\:}_{2}{s}_{2}+\cdots\:{+\beta\:}_{n}{s}_{n}+\epsilon\:$$

Where $\:g$ indicates the dependent variable of the target values, $\:{\beta\:}_{0}+{\beta\:}_{1}+{\beta\:}_{2}\dots\:{+\beta\:}_{n}$ represents the coefficients of the standard linear regression, $\:{s}_{1}+{s}_{2}+\dots\:+{s}_{n}$ signifies the independent variables of the features and $\:\epsilon\:$ indicates the error term of the standard linear regression.

#### Step 2

LASSO introduces a penalty term based on the absolute values of the coefficients. This term, multiplied by a tuning parameter (λ), discourages large coefficients. The L1 Regularization equation for this case is as follows.

3 $$\:{L}_{1}=\lambda\:*\left(\left|{\beta\:}_{0}\right|+\left|{\beta\:}_{1}\right|+\left|{\beta\:}_{2}\right|\dots\:+\left|{\beta\:}_{n}\right|\right)$$

#### Step 3

LASSO aims to minimize the sum of squared errors between predicted and actual values while also minimizing the L1 penalty term.

#### Step 4

By incorporating the L1 penalty, LASSO shrinks coefficients towards zero. When λ is large enough, some coefficients become zero, effectively removing those variables from the model.

#### Step 5

The choice of λ is crucial. A larger λ leads to more coefficients being driven to zero, while a smaller λ allows more variables to have non-zero coefficients. LASSO regression offers a powerful approach for both prediction and feature extraction, especially valuable for high-dimensional datasets with many features. Therefore, using the LASSO regression algorithm, clustering is done as follows.

Our dataset contains 64 EEG channels, each with 2560 samples, resulting in a total of 163,840 data points. LASSO regression is employed to reduce the dimensionality of these signals. By applying LASSO, we achieve a tenfold reduction in the number of features per channel, leading to a compressed representation with 256 features per patient.

**Fig. 2:**
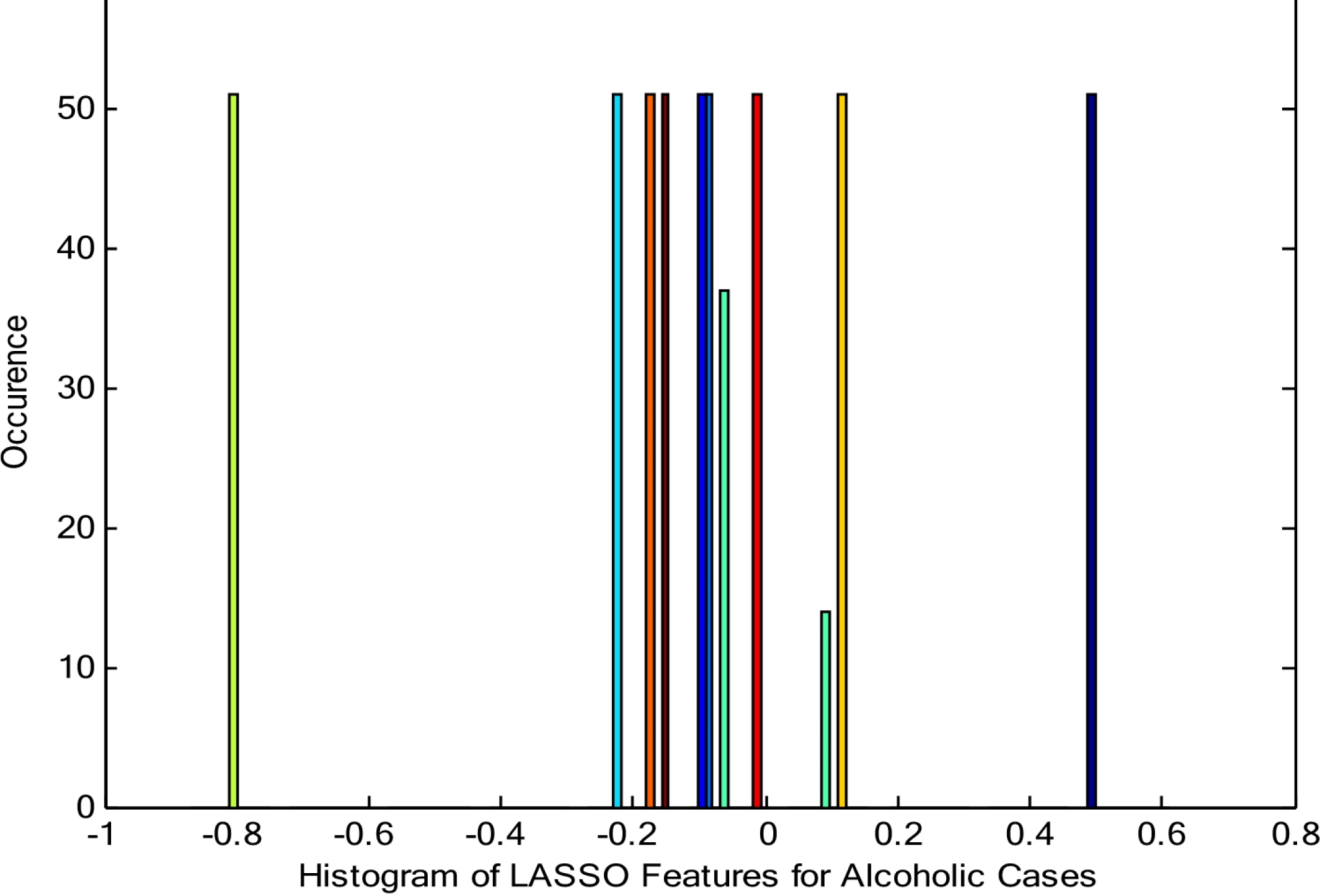
Histogram of the LASSO regression features for alcoholic EEG cases.

In this case, the parameter of regularization λ = 0.5. To assess whether LASSO regression preserves the inherent non-linearity of the EEG signals, we analyze the distribution of the resulting features using a histogram plot (Fig. 2). A histogram visually depicts the frequency of occurrence for different data values. Our analysis suggests that the non-linear dynamics in the alcoholic EEG signals are reflected in the non-normal distribution observed in the histogram. Table 1 summarizes the average values of key statistical parameters (mean, variance, skewness, and kurtosis) calculated from the LASSO regression features for alcoholic EEG signals.

**Table 1:** Average values of key statistical parameters of LASSO regression features for alcoholic patients.

S.No	Statistical Parameters	Values
1	Mean	− 0.0748
2	Variance	0.0001
3	Skewness	0.0161
4	Kurtosis	− 0.9808
5	Entropy	− 3.1863
6	Energy	0.0256

Table 1 shows the average values of the LASSO regression features for alcoholic patients, the mean average value indicates the alcoholic signals are negative skewed, variance average value represents the alcoholic signals are consistent, skewness average value represents the alcoholic signals are right skewed and kurtosis average values shows the lighter tails than a normal distribution. Relative to the dataset, the negative entropy value shows that there is low uncertainty. This relates to the variance, which is quite small and indicates that the data points are closely grouped around the mean. The energy value of for compressed features per patient shows that low overall power is possessed by signal. This low energy value conforms with a small variance in data implying no large fluctuations in this signal and it keeps steady. Finally the LASSO regression features of alcoholic signals exhibit non-linearity; therefore, feature selection methodologies are utilized to further reduce the dimensionality of the signals. This helps to identify the most significant features for classification.

### Feature selection via different metaheuristic algorithms

After using the LASSO regression approach for clustering, a multitude of metaheuristic methods are used to choose the efficient features from the clusters. Particle Swarm Optimization (PSO), Binary Coding Harmony Search (BCHS), and Binary Dragonfly Algorithm (BDA) are the different metaheuristic algorithms that are examined here for feature selection.

### Particle swarm optimization (PSO)

Based on how birds behave in social groups, the PSO algorithm is a population-based search engine. PSO is computationally affordable, both in terms of speed and memory utilization, and only requires simple mathematical expressions^28^. Social interaction and learning from one another are the key components of PSO. Particles inside the swarm migrate to resemble their better neighbors depending on the knowledge they have acquired. Neighborhood development shapes the PSO’s organizational framework. It is possible for neighbors to speak with one another. The star topology, ring topology, and wheels topology are among the several neighborhood types that have been identified and investigated. In this paper, we employ the PSO algorithm which utilizes a global best strategy, as shown below^29^.

#### Step 1: Initialization

The algorithm begins by creating a swarm of particles, denoted by $\:Q\left(t\right)$. Each particle, represented by $\:{Q}_{i}\:\epsilon\:Q\left(t\right)$, has a position $\:{S}_{i}\left(t\right)$ randomly distributed within the search space (hyperspace) at the initial time step $\:(t\:=\:0)$.

#### Step 2: Fitness evaluation

The performance of each particle is then evaluated using its current position $\:{S}_{i}\left(t\right)$. This evaluation assigns a fitness score $\:F\left({S}_{i}\left(t\right)\right)$that reflects how good the particle’s position is in terms of solving the optimization problem.

#### Step 3: Update personal best

Each particle compares its current performance $\:{S}_{i}\left(t\right)$ to its best performance encountered so far (personal best):

if $$\:F\left({S}_{i}\left(t\right)\right)<\:{q}_{id}$$ then

i) $$\:{q}_{id}=\:F\left({S}_{i}\left(t\right)\right)$$
ii) $$\:{Q}_{i}=\:{S}_{i}\left(t\right)$$

If the current performance is better, the particle’s personal best position is updated.

#### Step 4: Update global best

All particles in the swarm can access information about the best performing particle found so far (global best). This allows the swarm to collectively learn and move towards promising areas of the search space.

if $$\:F\left({S}_{i}\left(t\right)\right)<\:{q}_{gd}$$ then

i) $$\:{q}_{gd}=F\left({S}_{i}\left(t\right)\right)$$
ii) $$\:{Q}_{g}={S}_{i}\left(t\right)$$

#### Step 5: Velocity update

Based on the personal best position and the global best position, the velocity vector of each particle is updated. This velocity vector determines the direction and magnitude of movement for each particle in the next iteration.

4 $$\:{\mathcalligra{v}}_{id}\left(t+1\right)=\omega\:{\mathcalligra{v}}_{id}\left(t\right)+{\eta\:}_{1}*rand\left(\right)*\left({q}_{id}\left(t\right)-{s}_{id}\left(t\right)\right)+{\eta\:}_{2}*rand\left(\right)*\left({q}_{gd}\left(t\right)-{s}_{gd}\left(t\right)\right)$$

In Eq. (4), the second term on the right-hand side represents the cognitive component, while the final term signifies the social component.

#### Step 6

Move each particle based on its updated velocity.

i) $$\:{s}_{id}\left(t+1\right)={s}_{id}\left(t\right)+{\mathcalligra{v}}_{id}\left(t\right)$$
ii) $$\:t=\left(t+1\right)$$

#### Step 7

Continue steps 2 through 5 until completion. A particle’s shifting velocity to return to the best solutions increases with the particle’s distance from both the global best location and its individual best solution to at this point.

### Binary coding Harmony Search (BCHS)

A novel meta-heuristic optimization technique called harmony search (HS)^30^ mimics the process of musical inventiveness, in which performers explore instrument pitches to discover the ideal harmonic phase. Since each variable’s possible values were limited to the numbers 0 and 1, the binary coding approach in HS took the role of the float encoding technique used in this investigation. The following is a description of the optimization challenge^30^:

5 $$\:Minimizing\:f\left(g\right)\:\:subject\:to\:{g}_{i}\,\epsilon\left\{\text{0,1}\right\}\:\:\:\:{g}_{i}\,\epsilon \,g,\:i=\text{1,2},\dots\:\dots$$

Where $\:f\left(g\right)\:\:$indicates the function of objective, g represents the decision variable each set $\:{g}_{i}$ and $\:n$ total number of decision variable. The following is an expression of the BCHS’s detailed implementation:

Step 1: The algorithm’s factors are being initialized. The factors encompass the harmony memory size (HMS) and the harmony memory consideration rate (HMCR).
Step 2: Setting up the harmony memory. Within the viable solution space, the HM represented by $\:HM={\left[{g}^{1}{g}^{2}\dots\:.{g}^{HMS}\right]}^{T}$ is initialized at random.
  6 $$\:HM=\left[\begin{array}{ccc}{g}_{1}^{1}&\:\cdots\:&\:{g}_{n}^{1}\\\: \vdots &\:\cdots\:&\: \vdots \\\:{g}_{1}^{HMS}&\:\cdots\:&\:{g}_{n}^{HMS}\end{array}\right]\left[\begin{array}{c}f\left({g}^{1}\right)\\\:\vdots\\\:f\left(\left({g}^{HMS}\right)\right)\end{array}\right]$$
Step 3: Making up renewed harmony. Using the randomization and harmony memory consideration rule, which are established by the pre-defined HMCR, a new harmony, designated as $\:{g}^{{\prime\:}}=\left({g}_{1}^{{\prime\:}},{g}_{2}^{{\prime\:}}\dots\:.{g}_{n-1}^{{\prime\:}},\:{g}_{n}^{{\prime\:}}\right)$ is created. Similar to the improvisation process, the pitch adjustment operator is eliminated in this investigation. The following is how the specific procedure is stated:
  7 $$\:{g}_{i}^{{\prime\:}}=\left\{\begin{array}{c}{g}_{i}^{{\prime\:}}\:\epsilon\left\{{g}_{i}^{1},{g}_{i}^{2},\dots\:{g}_{i}^{HMS}\right\};\:\:if\:U\left(\text{0,1}\right)\le\:HMCR\\\:{g}_{i}^{{\prime\:}}\epsilon\left\{\text{0,1}\right\};\:\:otherwise\end{array}\right.$$
  Where $\:{g}_{i}^{{\prime\:}}$ represents the candidate of New Harmony with $\:{i}^{th}$ element.
Step 4: The harmony memory being updated. When it exceeded the weakest one in the HM, the newly created $\:{g}_{i}^{{\prime\:}}$ took its position.
Step 5: Assessing the termination standard. The stopping condition must be met for the iterative search to end and the ultimate outcome to be produced. Should this not be the case, repeat steps (3) and (4).

### Binary Dragonfly Algorithm (BDA)

The best features from the retrieved features from the EEG signals have been chosen in the proposed study using the Binary Dragonfly Algorithm (BDA)^31^. Recent developments in metaheuristic swarm intelligence have led to the development of the Dragonfly algorithm, a successful solution to a number of continuous optimization issues, including the machine learning optimization problem, the localization problem in networks, and the economic emission dispatch problem. In alcoholic EEG signal categorization and feature reduction, BDA offers a strong incentive for its application. Enhancing the accuracy and efficiency of EEG-based alcoholic detection systems might be made easier with its competitive performance, flexibility to dataset characteristics, interoperability with binary-encoded alcoholic EEG data, and equal emphasis on exploration and exploitation. Exploration and exploitation are the two stages of the BDA that go into fixing any given issue. The BDFA is a straightforward algorithm that leads to faster convergence to optimal solutions with fewer parameters. An intrinsic feature of many optimization methods influenced by nature is the seeming unpredictability in the behavior of BDAs. By enabling the algorithm to investigate many solutions, it raises the probability of discovering globally optimum or nearly optimal solutions in intricate problem domains. Therefore, utilizing the binary form of the dragonfly method, the best feature selection from the alcoholic EEG signal feature space is characterized as a binary optimization issue in this study.

The response to the selection of features issue is represented as a vector of $\:1s$ and $\:0s$, where ‘0’ denotes that the relevant feature is not picked and ‘1’ denotes that it is. Equation (8) describes how the fitness parameter of the feature selection issue is represented using the efficiency of classification and a few chosen features^31^.

8 $$\:Fitness=\alpha\:{\gamma\:}_{R}\left(G\right)+\delta\:\frac{\left|S\right|}{\left|N\right|}$$

Where $\:\alpha\:$ indicates the interval of the fitness function $\:\left[\text{0,1}\right]$, $\:\delta\:=\left(1-\alpha\:\right)$, $\:{\gamma\:}_{R}\left(G\right)$ represents the error rate of the fitness function, $\:\left|S\right|$ signifies the number of selected features of the fitness function and $\:\left|N\right|$ indicates the total number of extracted features from the alcoholic EEG signals. The BDA pseudocode is as follows:

### BDA pseudocode

Step 1: Initialize the population.

Step 2: For each iteration.

Calculate each solution Fitness using Eq. (8)

Update the position.

Step 3: End For.

Step 4: Return the optimal solution.

Table 2 Summarizes the average values of key statistical parameters like t-test, Friedman statistic test, Mann-Whitney U test, Z-Score with corresponding p values are calculated from the PSO, BCHS and BDA features for alcoholic EEG signals.

**Table 2:** Average values of key statistical parameters of PSO, BCHS and BDA features for alcoholic EEG signals.

S.No	Statistical Parameters	Feature Selection		
		PSO	BCHS	BDA
1	t-statistic	0.4534	0.3105	**4.2096E-39**
	$\:p-value$	0.5553	0.8725	**0.00001**
2	Friedman statistic	6.06	0.8	**40**
	$\:p-value$	0.1947	0.9385	**0.00001**
3	Mann-Whitney U test	1268	1225.5	**0**
4	Z-Score	− 0.2142	0.4986	**8.7005**
	$\:p-value$	0.8337	0.6171	**0.00001**

The $\:p-value$ is a measure of the significance level that will state the chance of finding an effect as larger otherwise larger than observed in the sample given that null hypothesis is true. Generally the value of $\:p$ should be small as possible because when $\:p$ close to zero, the null hypothesis is rejected and vice versa. Typically if the$\:\:p-value$ is:

i. $\:p-value<\:0.05$ the finding is indexed as statistically significant hence rejecting the null hypothesis.
ii. $\:p-value>\:0.05$ level then the outcome said not to be statistically significant hence the null hypothesis stands as it is not rejected.

The t-test is a hypothesis testing done to compare the means of two samples in particular, the control and experimental samples. It is widely employed in establishing the presence or lack of a statistically significant difference between the two groups’ means. The t-test results in a t-statistic and a$\:\:p-value$; both of which are utilized in order to evaluate significance. Typically if the t-statistic value is:

i. The scale ranges varies between − 1 and 1 indicates the not significant.
ii. The scale ranges varies between 1 and 2 otherwise − 1 to − 2 represents the not statistically significant.
iii. The scale ranges varies between above 2 represents the highly statistically significant.

As seen in Table 2, the results for the t-statistic with corresponding$\:\:p-value\:$indicates that:

i. The average t-statistic value for PSO feature selection is 0.4534, and the associated$\:\:p-value\:is\:0.5553$, indicating that the correlation is not statistically significant.
ii. The average t-statistic value for BCHS feature selection is 0.3105, and the associated$\:\:p-value\:is\:0.8725$, representing that the correlation is not statistically significant.
iii. The average t-statistic value for BDA feature selection is 4.2096E-39, and the associated$\:\:p-value\:is\:less\:than\:0.00001$, signifying that the correlation is highly statistically significant.

Friedman statistic is non-parametric test used to compare the means of more than two samples in case of abnormal distribution of data. It is a variation of the Wilcoxon signed-rank test of related samples, developed for use in with more than two groups. This test is used to apply the rank-sum test to more than two related groups to identify the variations in the medial values. They are often used in repeated measures design, in which the same subjects are tested several times under various circumstances. It yields a chi-square statistic, which, in turn, is compared to a chi-square value of the respective degrees of freedom. If the calculated statistic is greater than the critical value, then the null hypothesis which states that mean of both the median values is same, is rejected which shows that the groups are significantly different. Typically if the Friedman statistic value is:

i. Friedman statistic $\:>\:10-15$ suggests that there are marked differences between the groups and hence the null hypothesis should be rejected.
ii. Friedman statistic $\:<\:5-6$ signifies that there is no difference between the groups and therefore the null hypothesis cannot be rejected.
iii. Friedman statistic varies between 0 and 1 indicates the weak significance.

As demonstrated in the Table 2 the results of the Friedman statistic test values indicates that,

iv. The Friedman statistic test for PSO feature selection is 6.06, and the associated$\:\:p-value\:is\:0.1947$, indicating that the correlation is not statistically significant.
v. The Friedman statistic test for BCHS feature selection is 0.8, and the associated$\:\:p-value\:is\:0.9385$, representing that the correlation is not statistically significant.
vi. The Friedman statistic test for BDA feature selection is 40, and the associated *p – value is less than* 0.00001, signifying that the correlation is highly statistically significant.

The Mann-Whitney U test is another non-parametric statistical tool employed in comparing two independent groups’ distributions. It is used mostly to test for the null hypothesis that there is a difference in the median value of two given sets. Typically if the U statistics value is:

i. U statistic smaller than 100 corresponds to a significant statistical difference between the groups with greater values in the group size.
ii. When the U value is large, for instance, any number greater than 200, it shows that the groups are significantly different and the group with a larger number of employees has larger U values.
iii. If U values close to the sample size, it shows that there is no significant difference between the two groups.

As seen in Table 2, the results for the Mann-Whitney U test indicates that:

i. Mann-Whitney U test for PSO feature selection is$\:\:\text{U}\:=\:1268$: This value is quite bigger, which normally means that the two groups differ a lot.
ii. Mann-Whitney U test for BCHS feature selection is$\:\:\text{U}\:=\:1225.5$: This value is almost equal to the total sample size that indicates that the two groups are almost similar and representing that the correlation is not statistically significant.
iii. Mann-Whitney U test for BDA feature selection is$\:\:\text{U}\:=\:0$: This value shows that both groups are independent to an extent that all values in a given group are much larger than the values in the other group and demonstrating a highly significant difference between the groups.

The Z-score in other terms is called as the standard score that measures the deviation of an entity from the mean of a normally distributed dataset. In general, when determining Z score value is when Z-score equal to zero is interpreted as the fact that the chosen observation is equal to the average, Positive Z-score is indicates the observation above the mean and positive Z-score with value greater than one $\:(+1)$ represents the more than one standard deviation above the mean and Negative Z-score is indicates the observation below the mean and negative Z-score with value less than minus one $\:(-1)$ represents the more than one standard deviation below the mean. As demonstrated in the Table 2, the results of Z-score value directs that:

i. Z-score for PSO feature selection is $\:-0.2142$ and the corresponding$\:\:p-value\:is\:0.8337$, indicating that the correlation is not statistically significant.
ii. Z-score for BCHS feature selection is $\:0.4986$ and the corresponding$\:\:p-value\:is\:0.6171$, indicating that the correlation is not statistically significant.
iii. Z-score for BDA feature selection is $\:8.7005$ and the corresponding$\:\:p-value\:is\:0.00001$, signifying that the correlation is highly statistically significant and extremely outlier values.

**Fig. 3:**
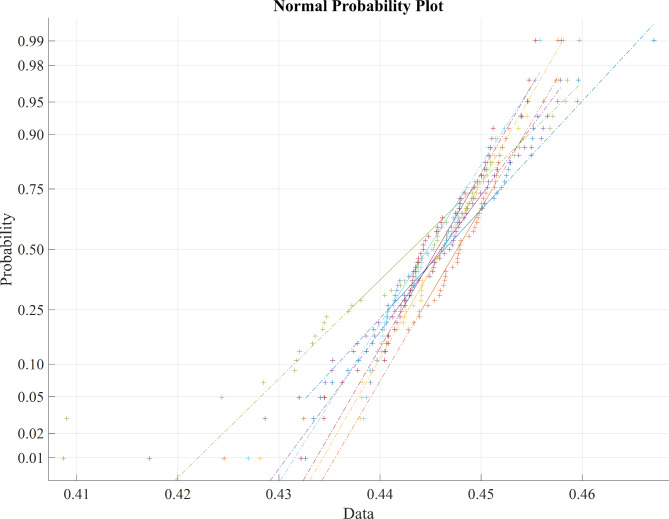
Normal probability distribution of the LASSO regression feature extraction based PSO features.

**Fig. 4:**
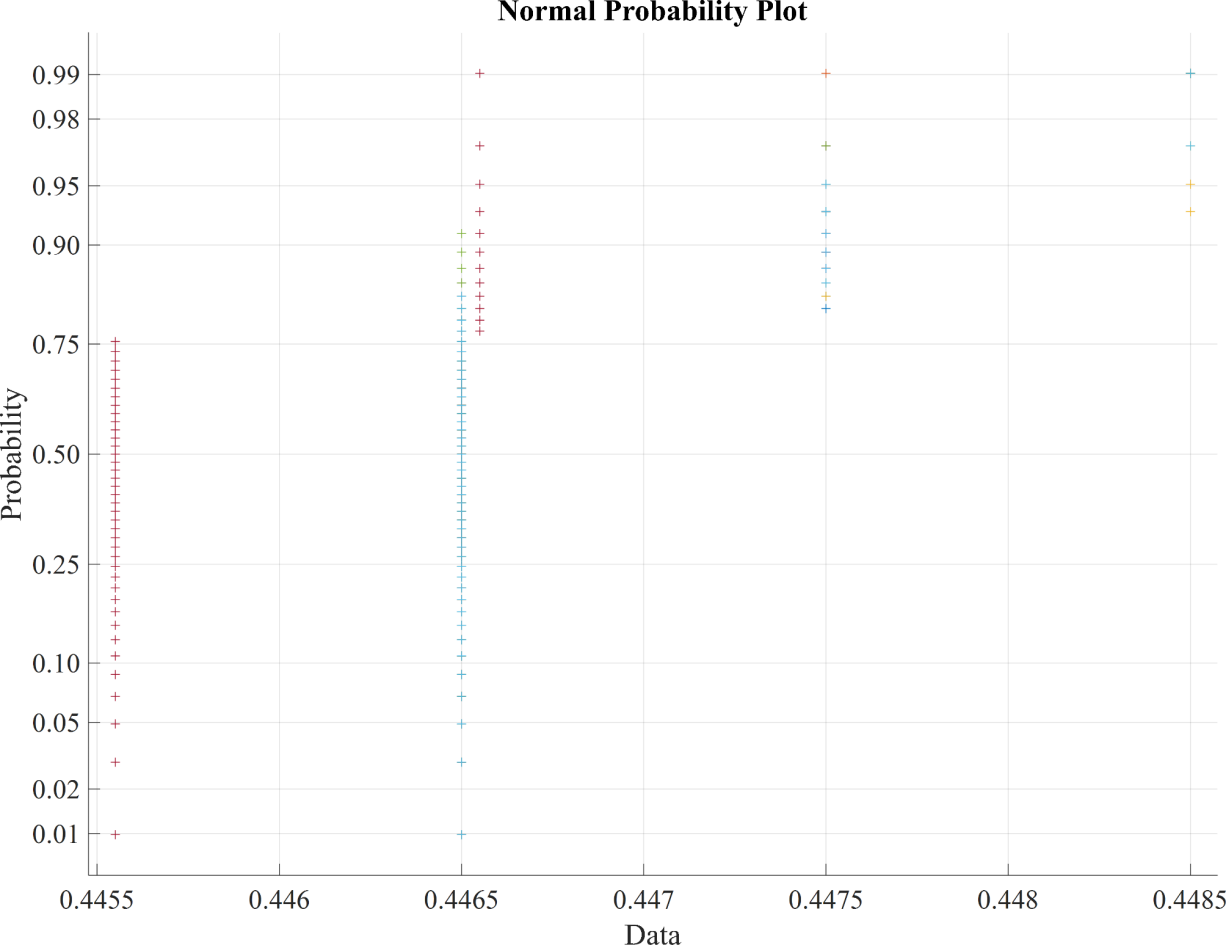
Normal probability distribution of the LASSO regression feature extraction based BCHS features.

**Fig. 5:**
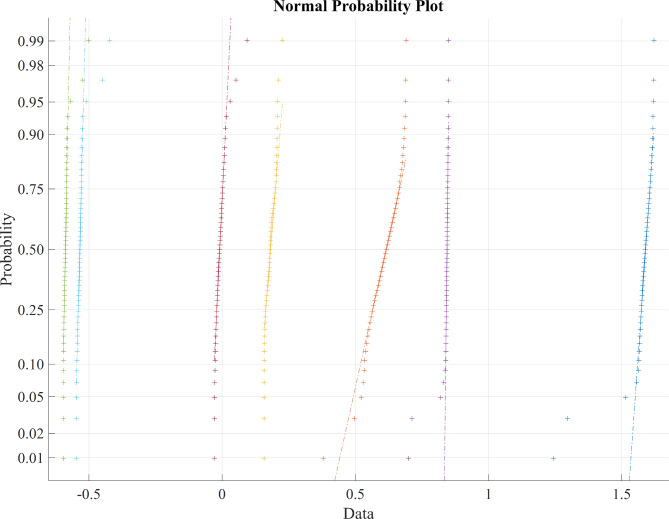
Normal probability distribution of the LASSO regression feature extraction based BDA features.

Table 2 presents the average statistical parameter analysis of various metaheuristic feature selection algorithms, revealing that the BDA algorithm outperforms other feature selection algorithms, yielding more significant results as evident from the average values of statistical parameters. Figure 3 represents the normal probability distribution of the LASSO regression feature extraction based PSO features, Fig. 4 displays the normal probability distribution of the LASSO regression feature extraction based BCHS features and the normal probability distribution of the LASSO regression feature extraction based BCHS features shows the Fig. 5.

The Normal probability distribution plot in Fig. 3, 4, 5 shows a clear relationship between the ‘PSO’, ‘BCHS’ and BDA’ features. Figure 3 represents normal probability distribution plot reveals non-linear patterns and substantial overlap, indicating a deviation from normality and suggesting that the data not be linearly separable. Figures 4 and 5 exhibits the outliers of the features. The data points cluster tightly around a value of 0.45, indicating a strong correlation. This makes it easy to choose a target value for our classification models. Because the central area of the plot is concentrated around 0.45, we can set this value as the target for our classifiers.

**Fig. 6:**
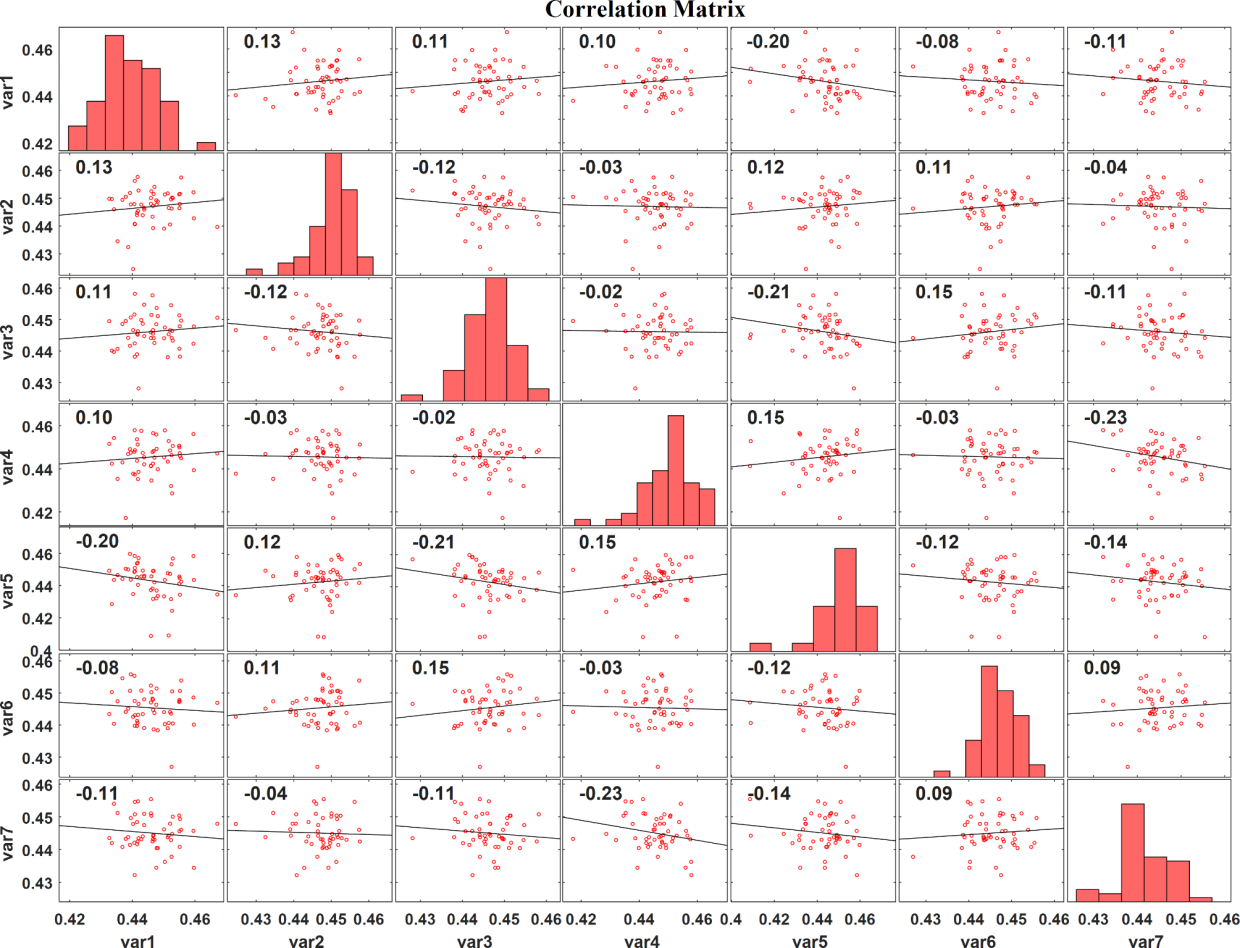
Correlation plot of the LASSO regression feature extraction based PSO features.

**Fig. 7:**
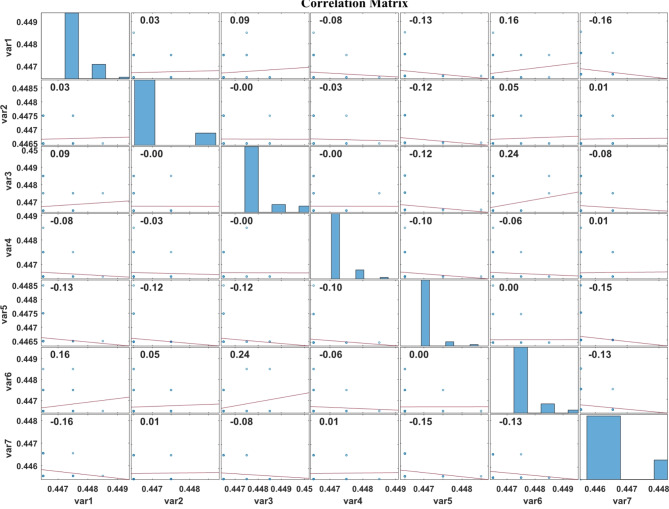
Correlation plot of the LASSO regression feature extraction based BCHS features.

**Fig. 8:**
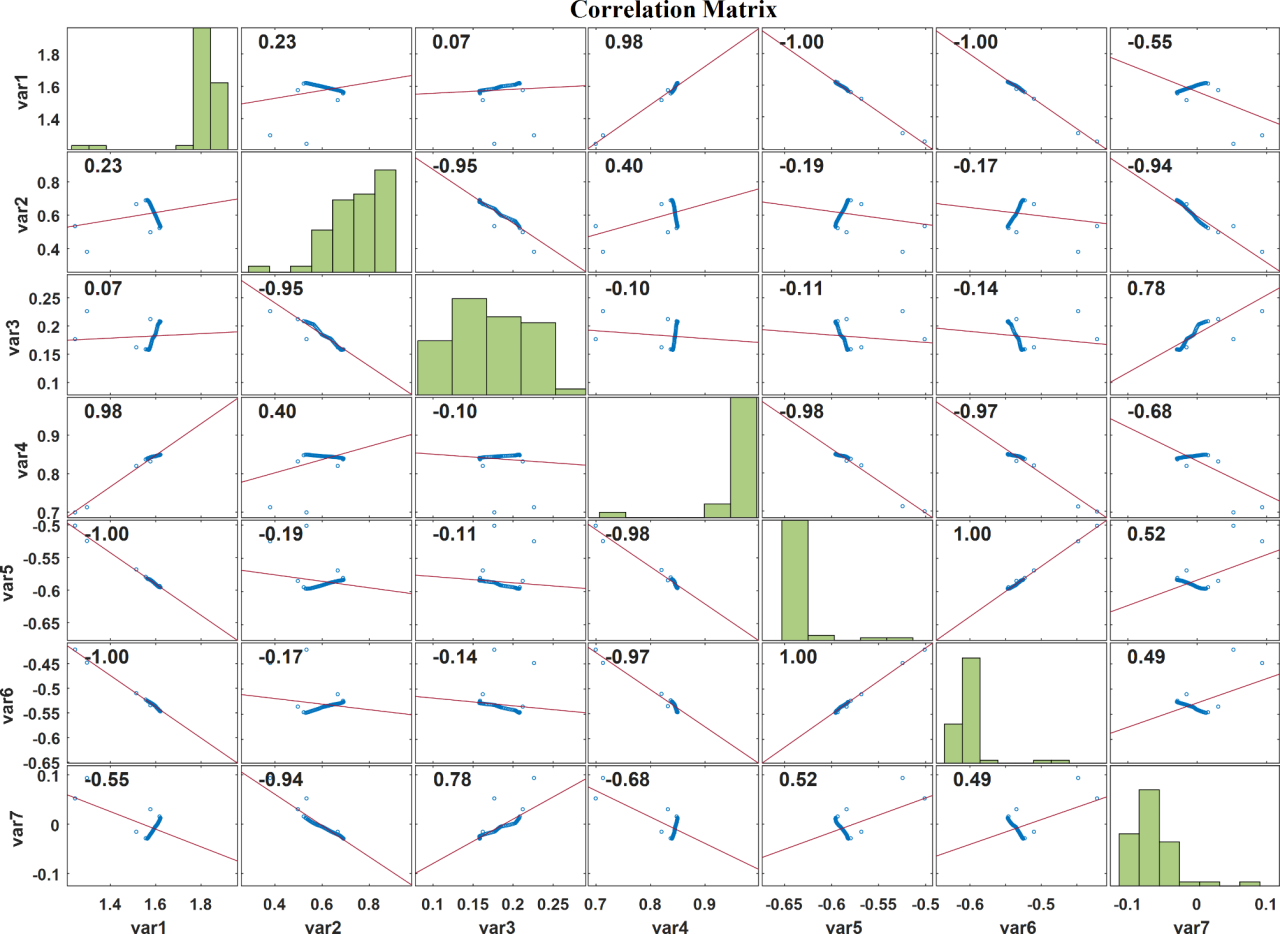
Correlation plot of the LASSO regression feature extraction based BDA features.

Figure 6 represents the correlation plot of the LASSO regression feature extraction based PSO features, Fig. 7 displays the correlation plot of the LASSO regression feature extraction based BCHS features and the correlation plot of the LASSO regression feature extraction based BCHS features shows the Fig. 8. A correlation plot demonstrates how multiple variables relate to one another. In this correlation plot visualization, there are scatter plots for the pairs of variables, histograms for the individual variable, and correlation coefficients showing the intensity as well as the direction of linear relationships. In this case, the correlation coefficients vary from − 1 to 1, where − 1 indicates the perfect negative correlation and + 1 represents the perfect positive respectively.

In Fig. 6, the variable histograms show some variability, ranging slightly above BCHS. The scatter plots reveal low positive and negative linear correlations between variable pairs. Significant correlations include var3 and var5, which have a correlation coefficient of -0.21, while var1 and var5 have one of -0.20. Overall, the variables show moderate variation and generally have weak correlations.

The histograms in Fig. 7 depict narrow distributions for each variable (var1 to var7). The scatter plots indicate weak linear relationships between variable pairs, with correlation coefficients ranging between − 0.16 and 0.24. Important correlations are: var3 and var5 (0.24), var1 and var6 (0.16). Generally, there is little difference between variables while their correlations are weak.

As indicated on Fig. 8, within the − 0.55 to 1.00 range of correlation coefficients, the variables are significantly related linearly. Notable linear relationships are between var1 and var5 with a correlation coefficient of 1.00, var1 and var3 with a correlation coefficient of 0.98, and var2 and var3 with a correlation coefficient of − 0.95 respectively. Histograms show that there is moderate dispersion for each variable. For the most part, data points underscore both very strong decreases as well as increases in between the variables.

### Classification methodology

In this research employed several classification models to analyze the effectiveness of the chosen features. These models included Long Short-Term Memory (LSTM) networks and Enhanced Artificial Neural Networks (EANN). Additionally, in this paper investigated Support Vector Machines (SVM) with different kernel functions (linear, polynomial, and RBF), Random Forest and Artificial Neural Network for classification.

### Proposed models

An enhanced four-layer neural network and an LSTM model were developed for improved performance of the neural network architecture. Both models used Python to implement them and a comparative analysis was conducted based on performance benchmarks.

### LSTM

**Fig. 9:**
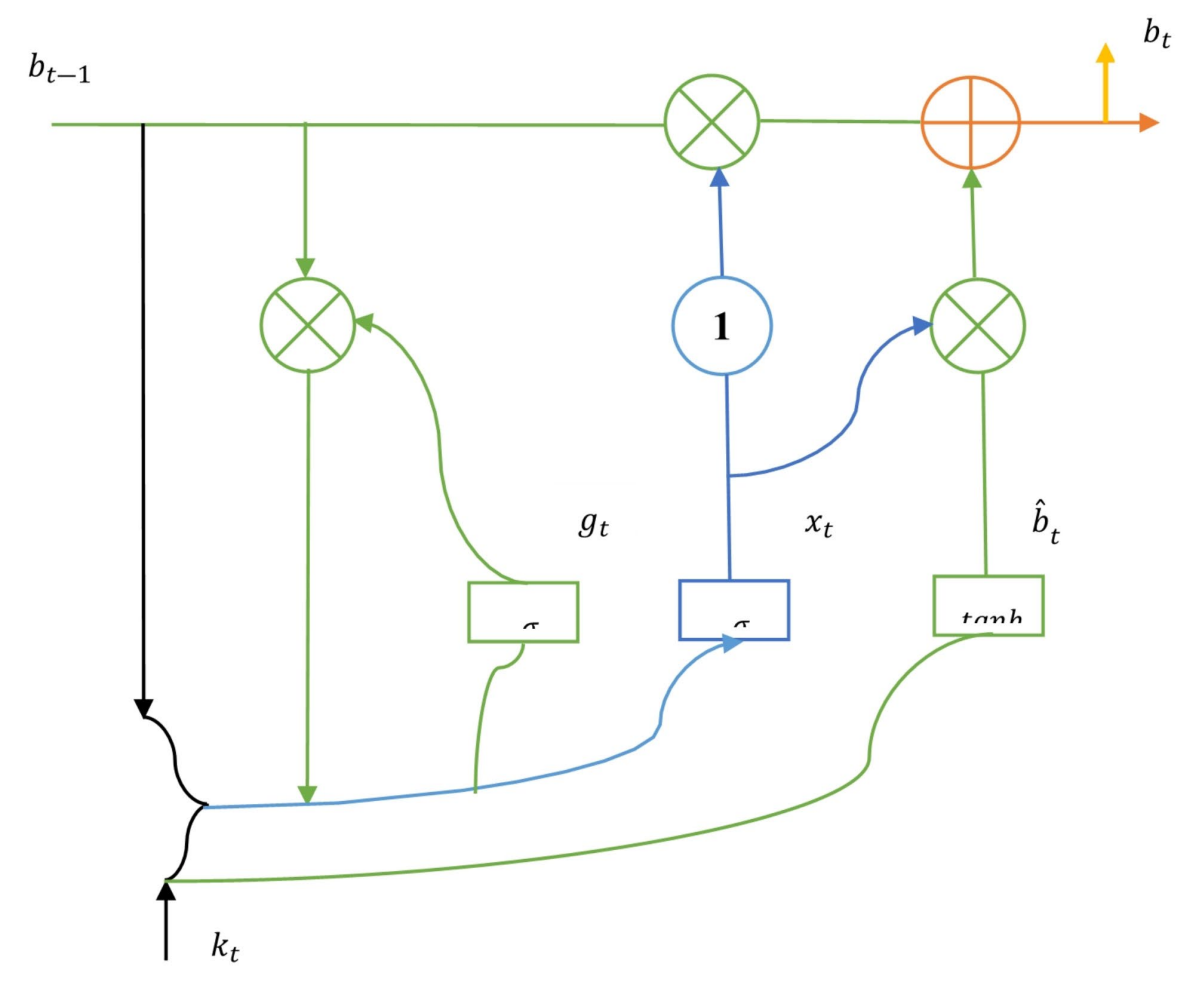
LSTM internal architecture.

Deep learning algorithms such as recurrent neural networks (RNNs) are the foundation of long short-term memory (LSTM)^32^. An external register or memory is not needed to save past outcomes since RNN is made up of recurrent structures that locally feed the firing ability. Due to the recurrent structures employed in RNN, LSTM has minimal complexity in computation. Figure 9 illustrates the internal architecture of the LSTM. The following operations are the foundation of how LSTM functions^33^.

9 $$\:{x}_{t}=\sigma\:\left({W}_{x}\cdot\:\left[{b}_{t-1},{k}_{t}\right]\right)$$

10 $$\:{g}_{t}=\sigma\:\left({W}_{g}\cdot\:\left[{b}_{t-1},{k}_{t}\right]\right)$$

11 $$\:{\widehat{b}}_{t}=tanh\left(W\cdot\:\left[{{g}_{t}*b}_{t-1},{k}_{t}\right]\right)$$

12 $$\:{b}_{t}=\left(1-{x}_{t}\right)*{b}_{t-1}+{x}_{t}*{\widehat{b}}_{t}$$

Where $\:\sigma\:$ indicates the sigmoid activation function, $\:tanh$ represents the activation function for hyperbolic tangent, $\:W$ indicates the input weights and connections of recurrent with either input gate, forget gate and output gate, $\:{b}_{t}$ signifies the new cell state and $\:{b}_{t-1}$ indicates the old cell state.

In an RNN, the learning process occurs in two phases such as structure learning and parameter learning. Nodes incorporate membership functions based on input variables, typically employing Gaussian functions defined by mean and variance. Single dimensional membership functions are assigned through spatial and temporal firing mechanisms. Structure learning involves determining the conditions under which rules are generated and activated, requiring firing strengths above a specified threshold usually between $\:0\:and\:1$ for each input. Parameter learning follows structure learning and aims to minimize the error cost function effectively.

### Enhanced artificial neural network

**Fig. 10:**
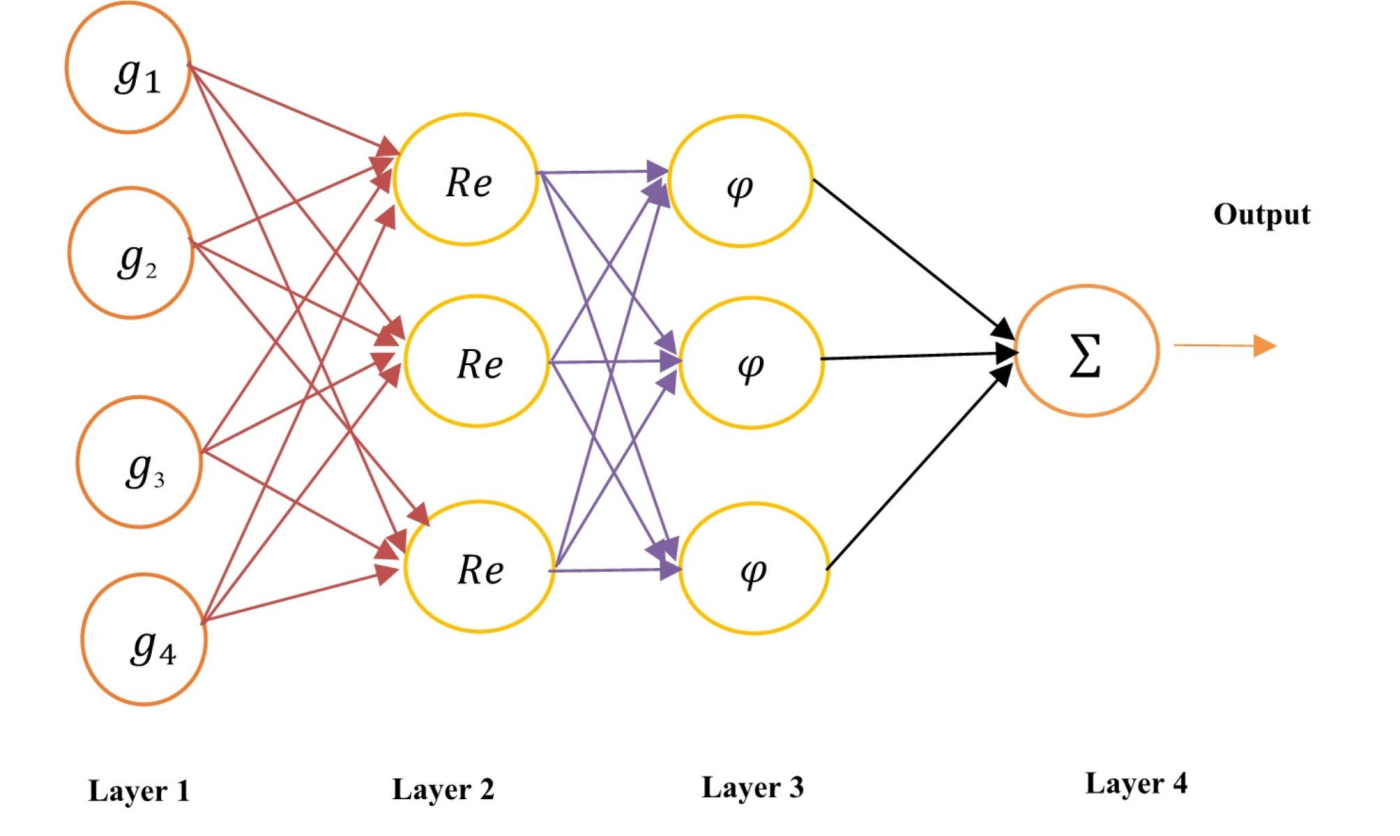
Architecture of the EANN.

A modernized four-layer design with ReLU activation, SeLU activation, ReLU activation in the first three layers and sigmoid in the last layer improves neural network effectiveness. It employs the Adam optimizer with a binary cross-entropy loss^34^. The number of hidden-layer neurons is not limited by any predefined limits. The network is completely linked, with weights and thresholds tuned. Prior to categorization, input is normalized. Tuning consists of four completely linked layers, with dropouts as needed. Kernels produce feature maps, whereas ReLU and radial basis procedures extract nonlinear features. Fully connected layers link each neuron to its neighboring layers. Our technique, which focuses on convolution layers and dropouts, surpasses LSTMs and standard neural networks. Pooling layers can be used to improve performance even more. Figure 10 illustrates the architecture of the EANN. The specifications of the EANN architecture components is shown in Table 3.

**Table 3:** Specifications of EANN architecture components.

S.No	EANN architecture components	Specifications
1	Number of Layers	4
2	Layer 1	ReLU
3	Layer 2	SeLU
4	Layer 3	ReLU
5	Layer 4	Sigmoid
6	Optimizer	Adam
7	Loss Function	MSE and Binary Cross Entropy
8	Epoch	100
9	Size of the batch	150

Preprocess the input data by normalizing its values, ensuring everything is on a similar scale before feeding it into the network. Optimize the network’s internal workings by adjusting weights and thresholds during training. These act like dials that control how the network processes information. Utilize a fully connected structure with four hidden layers. In each layer, all neurons are connected to all neurons in the next layer, allowing for complex information flow. Incorporate dropout layers at strategic points within the architecture. These temporarily remove some neurons during training, helping to prevent overfitting and improve generalization. Extract meaningful features from the data using convolutional layers. These layers apply filters (kernels) that slide across the input, identifying important patterns. Employ ReLU activation functions in the hidden layers. These functions introduce non-linearity, allowing the network to learn more complex relationships within the data. Unlike LSTMs and traditional neural networks considered, our architecture using only convolutional layers and dropouts achieves better performance in this specific case. While pooling layers were not used here, they could be further explored for potential performance improvements.

### Conventional models

The benchmarking process employed conventional models like ANN, Random Forest and SVM-RBF. The kernel methods in SVM-RBF give a good performance when it comes to classification, Random Forest uses decision trees that are based on ensemble learning while ANN’s major strength is its ability to recognize patterns in various neural network layers. These benchmarks were then used to compare the proposed model which performed better and gave more accurate results.

### Artificial neural network (ANN)

A computational model called an ANN classifier is modeled after the biological neural networks observed in the human brain. It is made up of networked components called neurons that cooperate to find solutions to certain issues, specifically those involving categorization. An input layer, an output layer, and perhaps several hidden layers comprise an ANN^35^. After implementing a weighted sum of inputs, the neurons in each layer employ a non-linear activation function. The output $\:q$ of a neuron $\:{s}_{q}$ in a hidden otherwise output layer can be calculated as follows^36^:

13 $$\:{s}_{q}=f\left(\sum_{p=1}^{n}{w}_{pq}{g}_{p}+{b}_{q}\right)$$

**Fig. 11:**
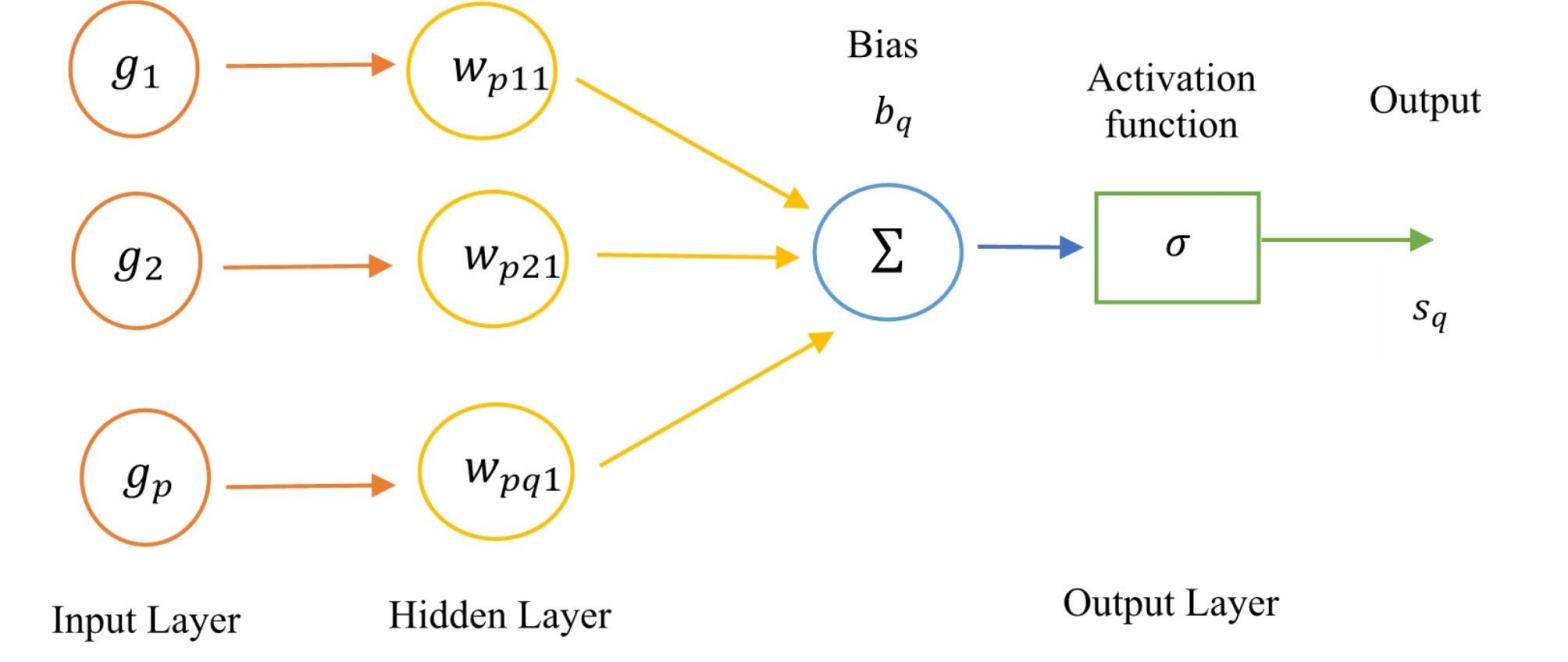
Architecture of the feed-forward neural network.

Where $\:{g}_{p}$ indicates the neuron’s input signals, $\:{w}_{pq}$ represents the weights of input $\:p$ and neuron $\:q$, $\:{b}_{q}$ indicates the bias of the neuron $\:q$, and $\:f\left(\cdot\:\right)$ indicates the type of activation function. In this case utilized the one hidden layer using ReLU activation and output layer using sigmoid activation function. Figure 11 illustrates the architecture of the feedforward neural network.

Through the use of a dataset, the ANN classifier is trained, and during this process, the weights $\:{w}_{pq}$ and biases $\:{b}_{q}$ are tuned in order to reduce the amount of classification error. This work employs an ANN-RBF network architecture comprising 32 input neurons in the input layer, 64 hidden neurons in the hidden layer and a single output layer, which collectively achieve a remarkably low Mean Squared Error (MSE) for both training and testing phases.

### Support Vector Machine (SVM)

Appropriate for both regression and classification applications, SVM is a sophisticated supervised machine learning technique^37^. The process identifies the feature-space hyperplane that best divides the classes. For both linear and non-linear categorization scenarios, SVM can use a variety of kernel functions such as linear, polynomial and gaussian. In terms of kernel functions, the most basic is the linear kernel. If classes can be divided into different groups by a straight line, the data can be classified as linearly separable. The linear kernel function and the input characteristics are combined linearly to form the decision boundary, which is expressed mathematically as follows^38^:

14 $$\:G\left(p,q\right)=p\cdot\:q$$

15 $$\:f\left(p\right)=w\cdot\:p+b$$

Where $\:p\:and\:q$ represents the input vectors, $\:w$ indicates the weight and $\:b$ represents the bias. For the linear kernel approach, an appropriate hyper parameter is selected through a random search scenario. The polynomial kernel thus adopts polynomial motives of the input characteristics to account for a more complex decision boundaries than offered by the linear kernel. In the case of non-linear data, it is appropriate. The polynomial kernel function characterized mathematically as follows:

16 $$\:G\left(p,q\right)={\left(p\cdot\:q+S\right)}^{d}$$

Where $\:S$ reflects a constant that balances the impact of higher-order components with that of lower-order ones, and d symbolizes the polynomial degree. In this case polynomial kernel is 1 is employed. The grid search approach is utilized in order to regulate the polynomial order while utilizing the polynomial kernel method. The gaussian kernel, which is often referred to as the RBF kernel, is a well-liked option for SVMs because of its efficiency to deal with non-linear data by locating input characteristics into an infinite degree space. The gaussian kernel’s mathematical expression is as follows:

17 $$\:G\left(p,q\right)=exp\left(-\gamma\:{||p-q||}^{2}\right)$$

A parameter called $\:\gamma\:$ determines how widely the kernel spreads and how much each training sample influences the system. In terms of the hyper parameter selection that is carried out for the Gaussian Classification algorithm, the gamma index of the Gaussian kernel is chosen from a range that begins at 0.2, continues through 0.4, reaches 0.6, and goes all the way up to 2.6. The testing process revealed that an MSE of 0.00000488 is attained at 250 iterations, with a corresponding gamma value of 2.0, indicating a significant reduction in error.

### Random Forest (RF)

In order to categorize the alcoholic signals, the feature values that are produced using distance metrics are placed as input into a classification algorithm^39^. This is done with the sole goal of categorizing the signals. In the majority of cases, the random forest classification methods are dependent on the categorization outcomes of a number of different tree models. Following that, each tree is given a random vector that is independent of the others and has the same distribution. Consequently, the training data and the randomly allocated vector give the tree the support it needs to carry out the classification. Using the 10-fold cross validation approach, the classification effectiveness is validated. The method is then assessed using the classification performance benchmarks. The random forest classifier $\:g\left(s\right)$ specified mathematically as follows^40^:

18 $$\:g\left(s\right)=majority\_\:vote\left({g}_{1}\left(s\right),{g}_{2}\left(s\right),\dots\:\dots\:{g}_{M}\left(s\right)\right)$$

Where $\:{g}_{p}\left(s\right)$ indicates the $\:pth$ decision tree prediction. The class that obtains the majority of votes from the various trees is the one that is ultimately selected as the output for classification challenges.

19 $$\:g\left(s\right)=\underset{C}{\text{argmax}}\sum_{p=1}^{N}\aleph\:\left({g}_{p}\left(s\right)=D\right)$$

Where $\:\aleph\:$ represents the function of indicator (true = 1; otherwise = 0) and the variable D represents the label of the classes.

### Evaluation scheme

Proposed model evaluation scheme is designed to fully test its performance, compare it with the existing conventional techniques and interpret the results by different performance benchmark measures. By this comprehensive assessment, we ensure that our model is robust and generalizable.

### Performance benchmarks analysis and model testing

Both, the proposed LSTM and the enhanced artificial neural network models were applied and tested using Python on a computer with 2 GHz processor and 16 GB RAM thereby proving its computational efficiency and accuracy. This research uses a step-by-step methodology to examine the EEG signals in classifying alcohol risk levels. In the beginning, feature extraction is done using LASSO regression method then feature selection involves a set of metaheuristic algorithms such as PSO, BCHS and BDA. After that classification is done through multiple classifiers. The performance of this approach is evaluated using various benchmark metrics such as Sensitivity$\:\:{S}_{e}$, Specificity $\:{S}_{p}$, Accuracy $\:{A}_{c}$, Matthews Correlation Coefficient $\:\left(MCC\right)$, Kappa Coefficient Analysis $\:\left(KCA\right)$, Mean Squared Error $\:\left(MSE\right)$, Good Detection Rate $\:\left(GDR\right)$, and Error Rate $\:{E}_{R}$. For reliable results 10-fold cross validation is conducted where dataset divided into 10 equal parts; each iteration uses 70% for training and remaining for testing which finally average performance metrics are calculated across all iterations. The Sensitivity$\:{\:S}_{e}$, Specificity$\:\:{S}_{p}$, Accuracy$\:{\:A}_{c}$, Matthews Correlation Coefficient$\:\:\left(MCC\right)$, Kappa Coefficient Analysis$\:\:\left(KCA\right)$, Good Detection Rate $\:\left(GDR\right)$, and Error Rate $\:{E}_{R}$ are obtained from the confusion matrix using the following formulas^41^:

20 $$\:{S}_{e}=\:\frac{TP}{TP+FN}*100$$

21 $$\:{S}_{p}=\:\frac{TN}{TN+FP}*100$$

22 $$\:{A}_{c}=\:\frac{TP+TN}{TP+TN+FP+FN}*100$$

MCC is a benchmark metric that is used to determine the performance of a binary classifier. The scale varies between$\:\:-1\:and\:1$. In this study $\:0.1\:to\:0.4$ indicates the worst prediction and $\:0.5\:to\:1$ represents the perfect prediction.

23 $$\:MCC=\:\frac{\left(TP\times\:TN\right)-\left(FP\times\:FN\right)}{\sqrt{\left(\right(TP+FP)}\times\:(TP+FN)\times\:(TN+FP)\times\:(TN+FN)}*100$$

KCA is a benchmark assessment statistic the extent of the agreement between two classes (alcoholic and normal) in binary classifier. It uses the amount of agreement that would be expected to occur by chance and gives a better estimate of agreement than does the percent agreement. The scale varies between − 1 and 1. In this study $\:KCA<0$ indicates the no agreement, $\:KCA\:=\:0.1\:to\:0.4$ indicates moderate agreement and $\:KCA\:=\:0.5\:to\:1$ represents the almost perfect agreement.

24 $$\:KCA=\frac{{A}_{c}-{E}_{a}}{1-{E}_{a}}$$

Where $\:{E}_{a}$ indicates the expected accuracy, which is expressed mathematically as follows,

25 $$\:{E}_{a}=\left(\frac{TP+FP}{N}\right)*\left(\frac{TP+FN}{N}\right)+\left(\frac{TN+FP}{N}\right)*\left(\frac{TN+FN}{N}\right)$$

26 $$\:GDR=\left(\frac{\left(TP+TN\right)-FP}{\left(TP+TN\right)+FN}\right)*100$$

27 $$\:{E}_{R}=\frac{FP}{(FP+TN)}*100$$

Where TP (True Positives) refers to the actual positive cases that have indeed been accurately predicted, TN (True Negatives) refer to such cases that are negative but were correctly predicted as such, FP (False Positives) means the wrongly predicted instances that belong to the positive class and FN stands for False Negatives, which are false-negative cases mistakenly predicted as negative.

28 $$\:MSE=\frac{1}{K}\sum_{g=1}^{K}\left({H}_{g}-{S}_{p}\right)$$

In this study, $\:{H}_{g}$ represents the observed EEG data values at a given time, while $\:{S}_{p}$ represents the target values for each of the $\:64$ models $\:(p\:=\:1\:to\:64)$. With a total of 122 observations per patient $\:\left(K\right)$, we utilized all distance features of the EEG data for both training and testing the classifiers. The training process entailed minimizing the Mean Square Error (MSE) values to the least amount of error. Interestingly, majority of classifiers obtained zero training error confirming to the hypothesis of maximum accuracy.

## Results and discussion

This section presents results from experiments, compares them with other conventional techniques, and then discusses these results. Table 4 presents the consolidated results of MSE values and confusion matrices for multiple classifiers using LASSO regression with PSO, BCHS and BDA feature selection techniques. According to Table 4, the LASSO regression with BDA-based EANN classifier achieved the lowest MSE of 1E-08, outperforming the other two models. In comparison, the LASSO regression with BCHS-based EANN classifier and PSO-based EANN classifier showed higher MSE values of 1.04E-06 and 8.125E-06, respectively. These results indicate that the LASSO regression with BDA-based EANN classifier performed best for alcoholic classification.

**Table 4:** Consolidated results of MSE values and confusion matrices for multiple classifiers using LASSO regression with PSO, BCHS and BDA feature selection techniques.

S.No	Classifiers	LASSO regression feature extraction														
		PSO					BCHS					BDA				
		TP	TN	FP	FN	MSE	TP	TN	FP	FN	MSE	TP	TN	FP	FN	MSE
1	LSTM	98	100	22	24	1.2665E-05	110	108	14	12	2.465E-06	117	113	9	5	5.3E-07
2	EANN	96	117	5	26	8.125E-06	114	111	11	8	1.04E-06	122	121	1	0	**1E-08**
3	ANN	89	108	14	33	1.314E-05	110	106	16	12	0.0000034	111	107	15	11	2.525E-06
4	Linear-SVM	84	85	37	38	3.4225E-05	81	111	11	41	2.3165E-05	101	90	32	21	1.57E-05
5	Polynomial-SVM	74	98	24	48	3.805E-05	88	103	19	34	0.00001642	103	98	24	19	1.049E-05
6	Gaussian-SVM	99	99	23	23	0.00001225	103	103	19	19	7.29E-06	107	105	17	15	0.00000488
7	RF	97	96	26	25	0.00001601	109	101	21	13	5.93E-06	107	101	21	15	6.925E-06

The consolidated result analysis of LASSO regression with PSO feature selection-based classifiers is presented in Table 5. Based on the results presented in Table 5, the proposed LASSO regression with PSO feature selection based EANN classifier achieved the accuracy of 87.30% with KCA of 0.7459. Table 6 shows the consolidated result analysis of LASSO regression with BCHS feature selection-based classifiers, while Table 7 details the results of LASSO regression with BDA feature selection-based classifiers. As observed from Table 6, the proposed LASSO regression with BCHS feature selection based EANN classifier achieved the accuracy of 92.21% with KCA of 0.8445. From the results presented in Table 7, the proposed LASSO regression with BDA feature selection based EANN classifier outperformed the accuracy of 99.59% with KCA of 0.9918. Based on the experimental study, optimized hyperparameters and best results of proposed LASSO regression with BDA feature selection method based ANN, LSTM and EANN classifiers are summarized in Table 8.

**Table 5:** Consolidated result analysis of LASSO regression with PSO feature selection-based classifiers.

S.No	Classifiers	Performance Benchmarks in %						
		*S*_*e*_	*S*_*p*_	*A*_*c*_	*MCC*	*KCA*	*GDR*	*E*_*R*_
1	LSTM	80.33	81.97	81.15	0.6230	0.6230	79.28	18.85
2	EANN	78.69	95.90	87.30	0.7572	0.7459	87.03	12.70
3	ANN	72.95	88.52	80.74	0.6223	0.6148	79.57	19.26
4	Linear-SVM	68.85	69.67	69.26	0.3853	0.3852	63.77	30.74
5	Polynomial-SVM	60.66	80.33	70.49	0.4180	0.4098	67.27	29.51
6	Gaussian-SVM	81.15	81.15	81.15	0.6230	0.6230	79.19	18.85
7	RF	79.51	78.69	79.10	0.5820	0.5820	76.61	20.90

**Table 6:** Consolidated result analysis of LASSO regression with BCHS feature selection-based classifiers.

S.No	Classifiers	Performance Benchmarks in %						
		*S*_*e*_	*S*_*p*_	*A*_*c*_	*MCC*	*KCA*	*GDR*	*E*_R_
1	LSTM	90.16	88.52	89.34	0.7870	0.7869	88.70	10.66
2	EANN	93.44	90.98	92.21	0.8445	0.8443	91.85	7.79
3	ANN	90.16	86.89	88.52	0.7709	0.7705	87.72	11.48
4	Linear-SVM	66.39	90.98	78.69	0.5919	0.5738	77.68	21.31
5	Polynomial-SVM	72.13	84.43	78.28	0.5699	0.5656	76.44	21.72
6	Gaussian-SVM	84.43	84.43	84.43	0.6885	0.6885	83.11	15.57
7	RF	89.34	82.79	86.07	0.7229	0.7213	84.75	13.93

**Table 7:** Consolidated result analysis of LASSO regression with BDA feature selection-based classifiers.

S.No	Classifiers	Performance Benchmarks in %						
		*S*_*e*_	*S*_*p*_	*A*_*c*_	*MCC*	*KCA*	*GDR*	*E*_R_
1	LSTM	95.90	92.62	94.26	0.8857	0.8852	94.04	5.74
2	EANN	**100**	**99.18**	**99.59**	**0.9918**	**0.9918**	**99.59**	**0.41**
3	ANN	90.98	87.70	89.34	0.7873	0.7869	88.65	10.66
4	Linear-SVM	82.79	73.77	78.28	0.5679	0.5656	75.00	21.72
5	Polynomial-SVM	84.43	80.33	82.38	0.6481	0.6475	80.45	17.62
6	Gaussian-SVM	87.70	86.07	86.89	0.7378	0.7377	85.90	13.11
7	RF	87.70	82.79	85.25	0.7058	0.7049	83.86	14.75

**Table 8:** Optimized hyperparameters best results of proposed LASSO regression with BDA feature selection method based ANN, LSTM and EANN classifiers.

S.No	Proposed Model	Layer	Activation Function	Topology	Dropout	Learning Rate	Epoch	Optimizer	Size of the batch	Loss Function	Accuracy in%
1	LASSO + BDA + ANN	2	ReLU + Sigmoid	32-64-100	0	0.001 to 0.5	500	Adam	250	MSE and Binary Cross Entropy	89.34
2	LASSO + BDA + LSTM	2	ReLU + Sigmoid	64-100-1	0.2	0.001 to 0.5	100	Adam	150	MSE and Binary Cross Entropy	94.26
3	LASSO + BDA + EANN	4	ReLU + SeLU + ReLU Sigmoid	64-100-50-32-1	0.2	0.0001 to 0.1	100	Adam	150	MSE and Binary Cross Entropy	**99.59**

**Table 9:** Conventional machine learning techniques hyperparameters.

S. No	Conventional Model	Hyperparameters
1	Linear-SVM	$\:\text{C}=1.0$
2	Polynomial-SVM	$\:\text{C}=5.0$ $\:\text{d}=5.0$
2	Gaussian-SVM	$\:{\upgamma\:}=2.0$
4	RF	$\:\text{T}\text{r}\text{e}\text{e}\text{s}\:\text{c}\text{o}\text{u}\text{n}\text{t}\:=\:125$ $\:\text{T}\text{r}\text{e}\text{e}\text{s}\:\text{d}\text{e}\text{p}\text{t}\text{h}\:\left(\text{m}\text{a}\text{x}\text{i}\text{m}\text{u}\text{m}\right)\:=\:75$ $\:\text{S}\text{p}\text{l}\text{i}\text{t}\text{s}\:\text{a}\text{n}\text{d}\:\text{l}\text{e}\text{a}\text{f}\text{s}\:\text{o}\text{f}\:\text{s}\text{a}\text{m}\text{p}\text{l}\text{e}\:\left(\text{m}\text{i}\text{n}\text{i}\text{m}\text{u}\text{m}\right)\:=\:04$

**Fig. 12:**
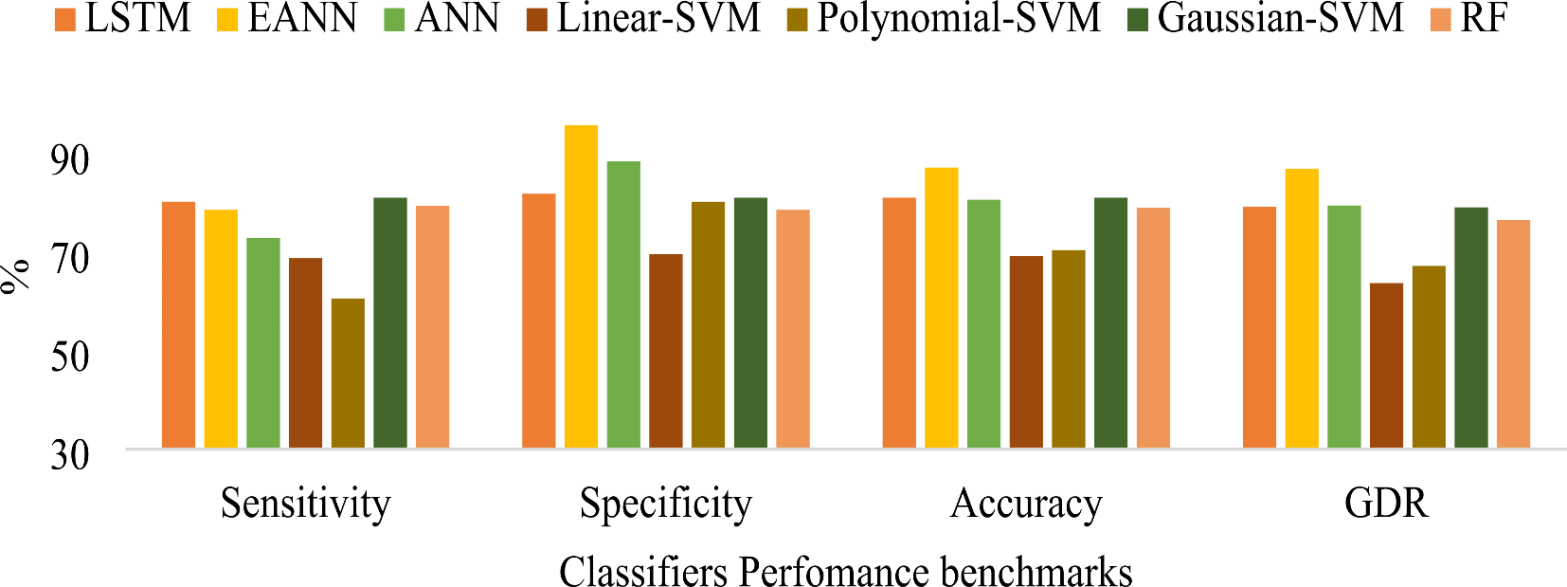
Graphical representation of consolidated result analysis for LASSO regression with PSO feature selection-based different classifiers.

**Fig. 13:**
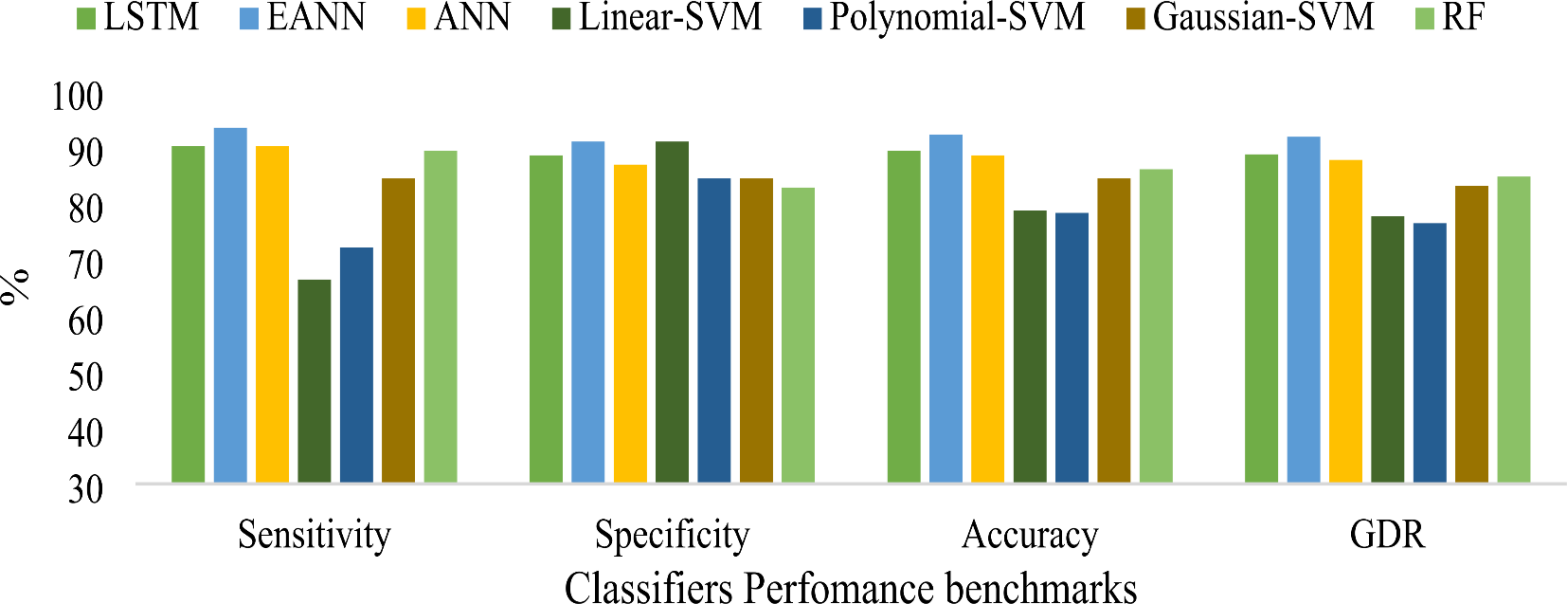
Graphical representation of consolidated result analysis for LASSO regression with BCHS feature selection-based different classifiers.

**Fig. 14:**
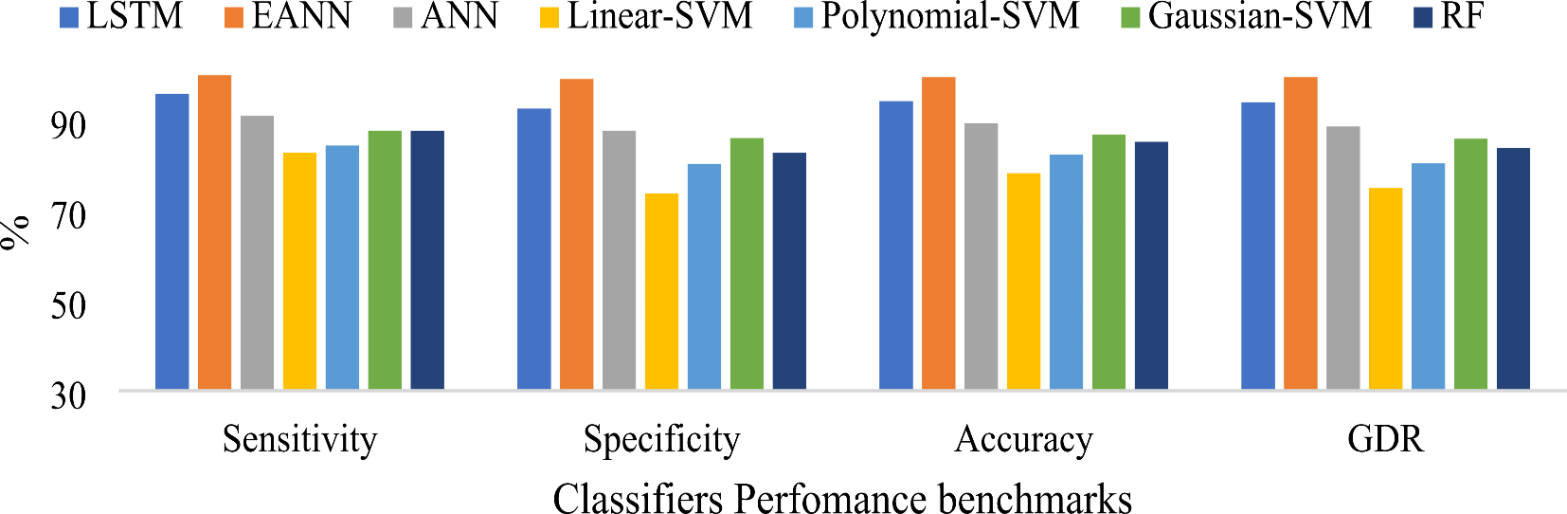
Graphical representation of consolidated result analysis for LASSO regression with BDA feature selection-based different classifiers.

According to Table 8, the EANN classifier combining LASSO regression with BDA feature selection achieved an outstanding accuracy of 99.59%, MCC of 0.9918, GDR of 99.59, KCA of 0.9918, error rate of 0.41 and Lower MSE of 1E-08, surpassing all other classification methods proposed in the study. Table 9 indicates the conventional machine learning techniques hyperparameters. Figure 12 represents the graphical representation of consolidated result analysis for LASSO regression with PSO feature selection-based classifiers. Figure 13 signifies the represents the consolidated result analysis of LASSO regression with BCHS feature selection-based classifiers. Figure 14 indicates the consolidated result analysis of LASSO regression with BDA feature selection-based classifiers.

**Fig. 15:**
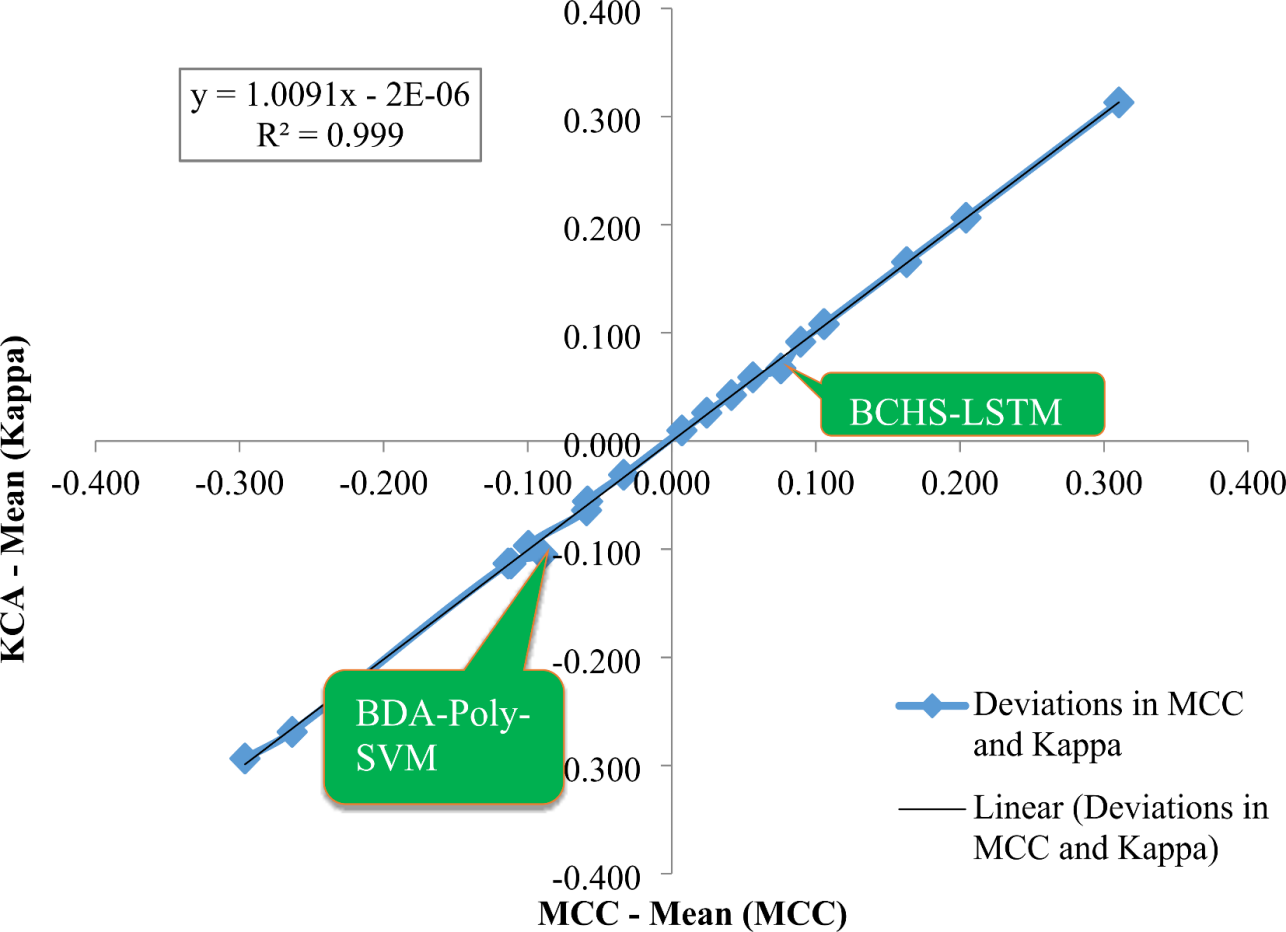
Performance analysis of classifiers using deviations MCC and KCA.

Figure 15 displays the exploration of the performances of proposed classifiers with the use of deviations of MCC and Kappa. As shown in Fig. 15, x-axis represents the deviation from the mean Kappa statistic (Kappa – Mean (Kappa)) and the y-axis represents the deviation from the mean Matthews Correlation Coefficient (MCC – Mean (MCC)) for the listed classifiers. The classifiers include: BDA-LSTM, BDA-EANN, BDA-ANN, BDA-Linear-SVM, BDA-Polynomial-SVM, BDA-Gaussian-SVM, BDA-RF, PSO-LSTM, PSO-EANN, PSO-ANN, PSO-Linear-SVM, PSO-Polynomial-SVM, PSO-Gaussian-SVM, PSO-RF, BCHS-LSTM, BCHS-EANN, BCHS-ANN, BCHS-Linear-SVM, BCHS-Polynomial-SVM, BCHS-Gaussian-SVM and BCHS-RF and also shows how well a classifier performs compared with other classifiers using a particular dataset. An upper right quadrant data point on this graph implies that the classifier was above mean for both Kappa Statistic as well as Matthews Correlation Coefficient; similarly, lower left quadrants show those instances where all measures represented are below average. There also appears to be linear regression line that passes through the dots is in the picture.

Given that the R² value on the regression line is approximately 0.999, it is an indicator that there is a positive and very strong association between whether the differences in Kappa occur in sync with differences in MCC or not. In other terms, the classifiers which are more capable than average classifying according to Kappa also happen to be more capable than average while classifying under MCC and vice versa. Based on Fig. 15, observed that both the BCHS-LSTM and the BDA-Poly-SVM classifiers are under the regression line which implies that they perform poorly when compared to other classifiers based on the Kappa statistic as well as the MCC. The comparison of our approach to some of the existing standard works with computational complexity and execution time in milliseconds (ms) regarding the automated detection of alcohol risk level is provided in Table 10.

**Table 10:** Comparison of our approach to some of the existing standard works.

Authors	Methodology	Accuracy (%)	Computational Complexity
Nandini et al.^9^	CNN + LSTM	91	$\:O\left({n}^{3}log\:n\right)$
Houchi and Lei^10^	DWT + CNNs and DWT + LSTMs	92.77 and 89	$\:O\left({n}^{2}log\:2n\right)$ and $\:O\left({n}^{3}log\:n\right)$
Leila et al.^11^	LSTM	93	$\:O\left({n}^{2}\right)$
Shrey Agarwal et al.^12^	Sliding Singular Spectrum Analysis based Independent Components analysis + ANN	97.37	$\:O\left({n}^{3}log\:2n\right)$
Rakhmatulin^13^	CNN	92	$\:O\left(n\:log\:n\right)$
Emad et al.^14^	MP-CNN	97	$\:O\left(n\:log\:n\right)$
Zhu et al.^15^	SVM	94	$\:O\left({n}^{3}\right)$
Hamid et al.^16^	DCNN + ReLU	98	$\:O\left({n}^{3}log\:n\right)$
Acharya et al.^17^	approximate and sample entropy, Lyapunov exponent and higher order spectra + SVM-Gaussian	91.7	$\:O\left({n}^{3}log\:n\right)$
Anuragi and Sisodia^18^	Flexibly Analytical Wavelet Transform + LS-SVM polynomial kernel	99	$\:O\left({n}^{2}log\:2n\right)$
**In this work**	LASSO + BDA + ANN	89.34	$\:O\left({n}^{2}log\:n\right)$
	LASSO + BDA + LSTM	94.26	$\:O\left({n}^{3}log\:2n\right)$
	LASSO + BDA + EANN	**99.59**	$\:O\left({n}^{4}log\:2n\right)$

Compared our approach to existing standard methods in Table 10 in terms of accuracy, computational complexity as well as execution time in milliseconds (ms). Also, it should be noted that our LASSO regression feature extraction combined with BDA feature selection and EANN classification have 99.59% of accuracy, $\:O\left({n}^{4}\:log\:2n\right)$ highest computational complexity which resulted in 450 ms execution time.

## Conclusion

This research employs non-linear methods of bio signal processing and pattern classification to estimate the alcoholic risk levels. It consists of the first step of clustering using LASSO regression and the second step of applying metaheuristic algorithms to reduce the feature space. The selected features are then classified using two proposed classifiers, namely EANN and LSTM, along with five benchmark classifiers. The findings show that a high level of classification accuracy of 99.59% when using the LASSO regression and BDA for feature selection together with the EANN classifier. In this systematic methodology, we reveal high performance in metaheuristic algorithms and classifiers of several kinds. The possible research directions include investigating different models of CNN and various types of multi-stage LSTM structures for the further improvement of the deep learning solutions for Alcoholic EEG signal classification.

## Data Availability

The datasets used in this study are publicly available online at: https://archive.ics.uci.edu/dataset/121/eeg+database.
